# A master equation approach to actin polymerization applied to endocytosis in yeast

**DOI:** 10.1371/journal.pcbi.1005901

**Published:** 2017-12-14

**Authors:** Xinxin Wang, Anders E. Carlsson

**Affiliations:** 1 Department of Bioinformatics, UT Southwestern Medical Center, Dallas, Texas, United States of America; 2 Department of Physics and NSF Center for Engineering MechanoBiology, Washington University, St. Louis, Missouri, United States of America; Northeastern University, UNITED STATES

## Abstract

We present a Master Equation approach to calculating polymerization dynamics and force generation by branched actin networks at membranes. The method treats the time evolution of the F-actin distribution in three dimensions, with branching included as a directional spreading term. It is validated by comparison with stochastic simulations of force generation by actin polymerization at obstacles coated with actin “nucleation promoting factors” (NPFs). The method is then used to treat the dynamics of actin polymerization and force generation during endocytosis in yeast, using a model in which NPFs form a ring around the endocytic site, centered by a spot of molecules attaching the actin network strongly to the membrane. We find that a spontaneous actin filament nucleation mechanism is required for adequate forces to drive the process, that partial inhibition of branching and polymerization lead to different characteristic responses, and that a limited range of polymerization-rate values provide effective invagination and obtain correct predictions for the effects of mutations in the active regions of the NPFs.

## Author summary

Endocytosis is a dynamic process by which cells internalize substances from outside the cell. Especially in yeast, endocytosis is mechanically demanding due to the high pressure difference across the cell membrane, or turgor pressure. Polymerization of a branched actin network is the major process providing the mechanical force to overcome the turgor pressure. Understanding the kinetics of the actin network, and the mechanical interaction between the actin network and the cell membrane, is thus crucial for the study of endocytosis. We develop an efficient mathematical framework for actin dynamics that can realistically incorporate these two features, thus providing a practical method for quantitatively modeling actin dynamics during endocytosis. The resulting model mechanistically reveals that spontaneous nucleation at the center of the endocytic site is required for successful endocytosis, distinguishes the roles of branching and polymerization, and predicts several other experimentally testable outcomes. The accuracy and efficiency of the method, in describing both mechanics and chemistry, render it applicable to a broad field of membrane-bending processes.

## Introduction

Forces exerted by polymerization of monomeric actin (G-actin) into filamentous actin (F-actin) are crucial for bending the cell membrane in many important cellular processes, including cytokinesis, cell migration, and, under some conditions, endocytosis [1]. Specifically, actin is required for yeast clathrin-mediated endocytosis (CME), a central mechanism that controls cellular signaling, nutrient uptake and membrane recycling [2]. CME is driven by a transient protein patch, in which different proteins appear in a well-defined sequence [3], including actin and its nucleators. The actin patch bends a small portion of the cell membrane into a highly curved invagination that encloses extracellular substances. The invagination is later severed and its contents, as well as lipids and membrane proteins, are released into the cytoplasm.

**Fig 1:**
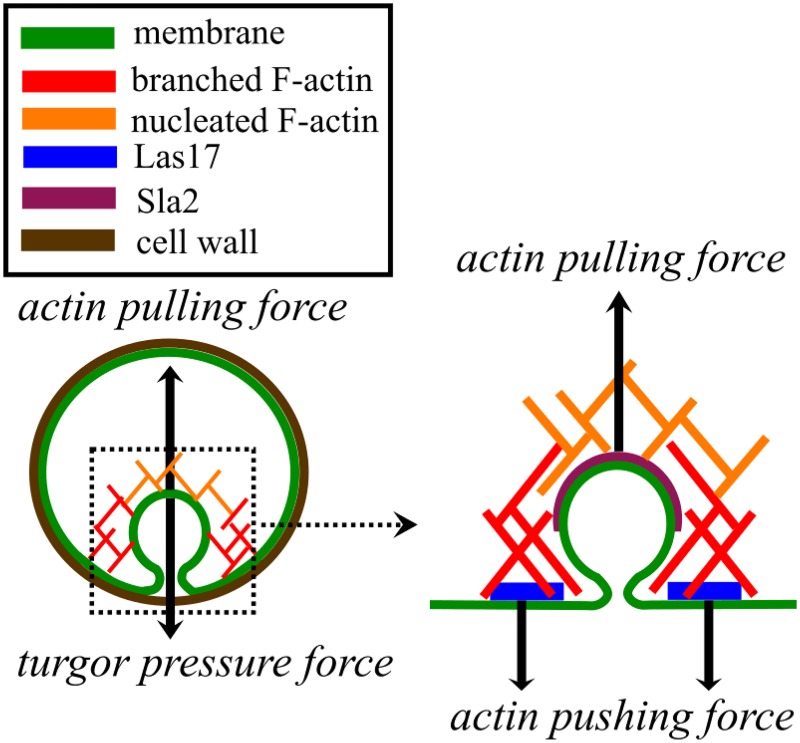
Model schematic. The membrane deformation is determined by a balance of forces between turgor pressure, the membrane bending energy, and actin pulling and pushing forces.

For this membrane-bending process, the actin network needs to exert both pulling forces and pushing forces (see Fig 1). The required pushing forces are several *pN* per filament [4, 5], mainly to overcome the large (∼0.2*MPa*) osmotic pressure difference [6] (turgor pressure) across the membrane, because of the small number (∼10^2^) [7] of actin filaments at each endocytic site. The machinery driving CME constitutes a coupled mechanochemical network [8]. Force regulates protein dynamics via processes such as the slowing of actin polymerization by opposing force; conversely polymerization of actin and assembly of curvature-generating proteins generate force. We are only beginning to understand the basic properties of this network.

Protein dynamics during CME have been extensively studied via fluorescence imaging methods. Assembly of endocytic proteins (EPs), including F-actin, was first quantified [3, 9] using relative fluorescence intensities. Later, Ref. [10] developed a method for measurement of the absolute counts of the EPs in fission yeast (*Schizosaccharomyces pombe*). In Ref. [7], and later in Ref. [11], absolute counts were measured in budding yeast (*Saccharomyces cerevisiae*). These studies have suggested a count of about 6000 polymerized-actin subunits at the endocytic site [2], with the counts of other proteins typically in the range of 50 to 300. Actin nucleators, or “NPFs”, precede actin polymerization, which proceeds over a period of about 15 seconds. These quantitative measurements have inspired several quantitative modeling studies of dynamics of the EPs [4, 8, 12, 13].

The mechanical aspects of CME are less well understood due to the difficulties of measuring forces on a scale of tens of nanometers *in vivo*. Balance of forces on the actin network requires that inward pulling forces at the center of the endocytic site are opposed by equal pushing forces from the outer regions of the actin network [4]. The mechanics of bending the cell membrane in CME were studied in detail in a recent model [5] based on the “Helfrich” free energy density [14]. The authors calculated the energy-minimizing shape functions of the membrane during endocytosis, using parameters fitted to electron microscopy tomography data [15]. However, the dynamics of the actin force were not included when obtaining the shape functions, so it was not possible to calculate a time-dependent shape nor to include the mechanochemical feedbacks driving the protein and shape dynamics. In Ref. [4], one of us treated actin as an actively growing gel simulated using a finite element method (FEM), and thus predicted endocytic invagination dynamics. However, the actin growth was modeled with a simple phenomenological description, which was not quantitatively compared to experiment. It is thus unclear how well the growing gel represents the actual dendritic structure of the network [16]. The mechanics of stationary CME membrane profiles were also treated in Refs. [17] and [18], but again neither treated the protein dynamics in the process.

A mechanochemical model of CME was proposed in Ref. [8]. It contained several types of feedback interactions, which indirectly impacted actin polymerization. This model explained several traits of endocytic mutants. However, the treatment of actin polymerization was highly simplified, and the model used an extremely low value of the turgor pressure.

A major challenge in developing a complete description of the mechanochemical network driving CME is to accurately model the actin network and its interaction with the cell membrane. In Ref. [12], we and others developed a stochastic model of actin polymerization during CME. The F-actin in the model was modeled via a stochastic-growth method that gave an explicit three-dimensional actin network, with parameters fitted to experimental data. The force opposing actin polymerization was assumed to “kick in” when the network reached a certain size. This work revealed some important feedback mechanisms between actin and its nucleators, required for CME. But the membrane mechanics were oversimplifed by using a step-function force opposing actin polymerization, and the membrane profile was not obtained explicitly. Additionally, this stochastic model required a large ensemble of repeated calculations to produce meaningful results. It was thus difficult to quantitatively fit the model, for use in other potential applications. Furthermore, stochastic models are difficult to use in studying possible oscillatory behaviors and bifurcations of the actin network that require the recognition of subtle changes in the F-actin count.

Deterministic rate-equation approaches, as in Ref. [19] and parts of Ref. [12], would thus be more convenient for experimental fitting and capturing subtle effects. However, such rate equations cannot treat the mechanics and geometry of the actin network because they describe only the average F-actin count rather than the spatial distribution of F-actin. A number of deterministic reaction-diffusion approaches have improved on rate-equation approaches by modeling the spatial distribution of F-actin explicitly, using various assumptions about dynamics and the interaction of F-actin with the cell membrane [20–28]. However, such methods have not explicitly included the oblique branched geometry of the network, and their force-generation component has not been validated by comparison to stochastic-simulation results.

Ref. [29] developed a two-dimensional treatment of the actin network from a spatially dependent rate equation, explicitly treating branching angles. The mesh size was small in comparison with the size of the cell, but still large enough to treat the coarse-grained density of F-actin. This method was shown to give promising results for global cell properties such as migration. However, it is not clear to what extent it can be applied to processes such as CME, which are fully three-dimensional and have significant structure at very small length scales.

In this paper, we propose a Master Equation (ME) method to treat the reciprocal interactions of polymerizing actin and its nucleators with a bending membrane, and apply it to CME. The ME method describes the spatial distribution of F-actin using a single simulation for a given set of parameter values, while having nearly the realism of the stochastic-growth approach implemented with a large numbers of runs. The methodology explicitly includes the branching geometry, and is validated by comparison with stochastic simulations for the case of an actin network pushing an obstacle. It uses a mesh size smaller than the characteristic size of the actin network, to calculate a probability distribution function (pdf) of actin subunits at given points in time and space. It builds on the work of Ref. [29] by treating a three-dimensional geometry, using a smaller mesh size (about 2 nm vs. 100 nm) that allows better treatment of actin-based forces, and calculating the pdf rather than the coarse-grained actin density. It differs from the reaction-diffusion approaches above in its more complete description of both the orientation and length of new branches in three dimensions, in its more accurate treatment of force generation, and in the use of a pdf. We apply the ME method to a mechanochemical model of CME in budding yeast that treats the time courses of F-actin, its nucleator Las17, and the deformation of the membrane. The model integrates the chemical variables F-actin and Las17 (slow), and the membrane shape variables (fast) into one dynamically interacting system, and shows how the actin network bends the cell membrane in real time.

The model accomplishes several important goals: 1) a theory of dendritic actin polymerization that is mechanistically realistic, numerically accurate and computationally efficient, 2) a mechanochemical model of the dynamics of the cell membrane driven by the actin network during CME, and 3) a more accurate model of CME that quantitatively determines several core parameters that were “floating” (not determined by experimental data) in the previous model [12]. The results of the ME model are consistent with experimental data [7, 12] for protein dynamics and the effects of mutations. New predictions from the model include the following: i) a spontaneous nucleation mechanism is required in the central portion of the endocytic site, ii) controlled inhibition of branching and/or polymerization lead to characteristic behaviors of the peak counts of actin and its main nucleator, and iii) a certain range of polymerization rates is required for robust invagination, and correct prediction of the peak counts of actin and its main nucleator in mutants.

## Methods

### Master equation (ME) approach

Our model treats dendritic actin network growth in the presence of capping, with new filaments created as branches induced by a planar distribution of nucleation-promoting factors (NPFs), as on a membrane or hard substrate. The model describes polymerization in three dimensions, but we introduce it in two dimensions first, for the sake of clarity. The dynamics of the F-actin probability distribution function *ρ* in the network are treated by a master equation including branching, spontaneous nucleation and severing:

$$\begin{array}{ccc} \frac{\partial \rho (x,y,t)}{\partial t} & = & \underset{\text{branching}}{\underset{︸}{\int_{0}^{l(y,t)}\frac{k_{br}(x,y+y^{\prime })}{2}N(t)[\rho (x-y^{\prime },y+y^{\prime },t)+\rho (x+y^{\prime },y+y^{\prime },t)]dy^{\prime }}}\\ & & +\underset{\text{nucleation}}{\underset{︸}{k_{nuc}(x,y,t)}}-\underset{\text{severing}}{\underset{︸}{k_{sev}\rho (x,y,t)}}. \end{array}$$ (1)

Here *k*_*br*_(*x*, *y*) is the branching rate constant, which gives the rate of branching per unit length of F-actin, per molecule of NPF in the membrane. The rate *k*_*nuc*_(*r*, *y*, *t*) describes the amount of actin generated per unit time by spontaneous nucleation, while the decay rate *k*_*sev*_ is assumed to be controlled by cofilin-driven severing; *l*(*y*, *t*) is the projection onto the *y*-axis of the length of the filaments added at each time step. The coordinate *x* is in the plane of the membrane, *y* is perpendicular to the membrane, and *t* is the time variable. We use a frame of reference in which the existing actin filaments are stationary, so convective terms are not required. *N*(*t*) is the number of molecules of the NPF on the membrane. We assume that the NPFs are uniformly distributed over the membrane, either because they diffuse rapidly in the membrane, or because their initial distribution is uniform. We do not explicitly treat the assembly of curvature-generating proteins. Rather, they are included as in Ref. [5], as a contribution to the forces acting on the membrane.

**Fig 2:**
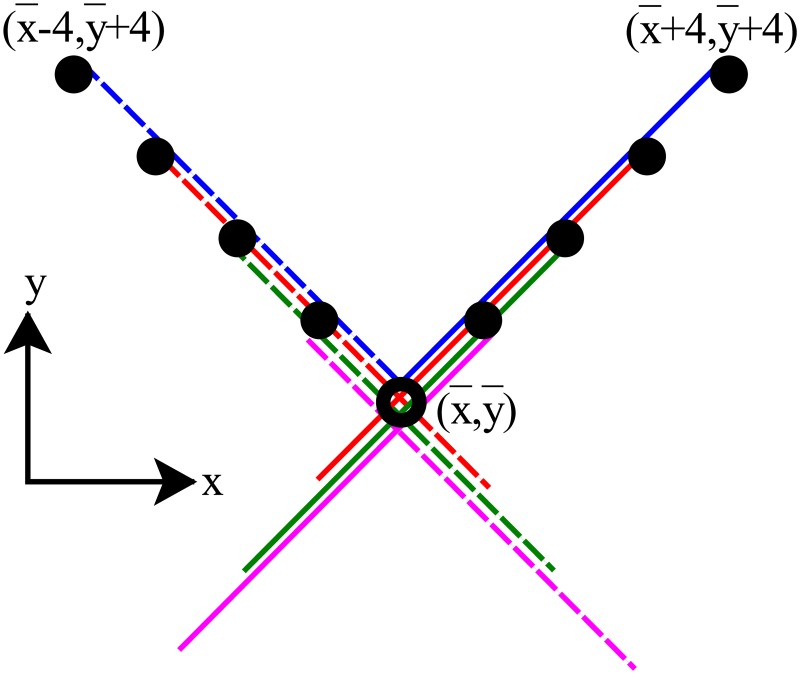
Schematic of discretized version of Eq 1 for the case of filaments having length four subunits (${\bar{l}}_{max}=4$). The subunits in solid circles can generate a branch of length $\bar{l}\left(\bar{y},t\right)$ that reaches the open circle $\left(\bar{x},\bar{y}\right)$. $\bar{x}$ and $\bar{y}$ are dimensionless coordinates normalized by $a/\sqrt{2}$, where *a* is the step size per added subunit. See Eq. S30 for definition of $\bar{l}\left(\bar{y},t\right)$.

In the model, we take all the filaments to have either a 45° or −45° angle with respect to the *y* direction, in line with the oblique alignment generally found in dendritic networks. New filaments instantly polymerize to a final length, whose projection on the *y*-axis is *l*(*y*, *t*) (the filament length multiplied by $\text{cos}\;45^{{}^{\circ}}=1/\sqrt{2}$). The length is determined by the force-dependent polymerization rate and the capping rate. We assume that capping (and thus the growth of a filament to its final length) occurs on time scales faster than the evolution of the invagination. The validity of this approximation is discussed below. At a given time, only new filaments that branch from certain F-actin subunits can increase *ρ*(*x*, *y*, *t*). These subunits are included in the integral in Eq (1). The two branching directions correspond to the *x* ± *y*′ and *y* + *y*′ terms in the integral in Eq (1). In practice, considering Fig 2 as an example, we use the dimensionless length $\bar{l}\left(y,t\right)=l\left(y,t\right)/\left(a/\sqrt{2}\right)$ normalized by the length of the actin monomer (*a*) projected onto the *y*-axis; $\bar{l}\left(y,t\right)$ is thus the number of subunits in a given new filament. We discretize Eq 1 accordingly (see Eq. S4) to describe the discrete spatial distribution of F-actin in the network. Fig 2 illustrates the case $\bar{l}\left(y,t\right)=4$ from Eq. S4. This two-dimensional version of the model could be applied to actin networks growing on a long strip of NPF, as in Ref [30].

We extend Eq (1) into three dimensions using a cylindrical coordinate system (see S1 Fig) The spatial coordinates become *r*, *θ* and *y*, and Eq (1) becomes

$$\begin{array}{ccc} \frac{\partial \rho (r,y,t)}{\partial t} & = & \frac{1}{2\pi }\int_{0}^{l(y,t)}dy^{\prime }k_{br}(r,y+y^{\prime })N(t)\int_{0}^{2\pi }\rho [R\left(r,\theta \right),y+y^{\prime },t]d\theta \\ & & -k_{sev}\rho (r,y,t)+k_{nuc}(r,y,t), \end{array}$$ (2)

where $R=\sqrt{{y^{\prime }}^{2}+r^{2}-2ry^{\prime }\text{cos}\theta }$ is the radial coordinate of the base of a branch having its end at radius *r*. Again, we discretize the time and spatial dependence, as described in Eq. S5.

More details of the simulation procedure are described in the Supplementary Material. This azimuthally symmetric ME approach is directly applicable to templated-nucleation experiments of the type described in Ref. [31].

### Method validation

**Fig 3:**
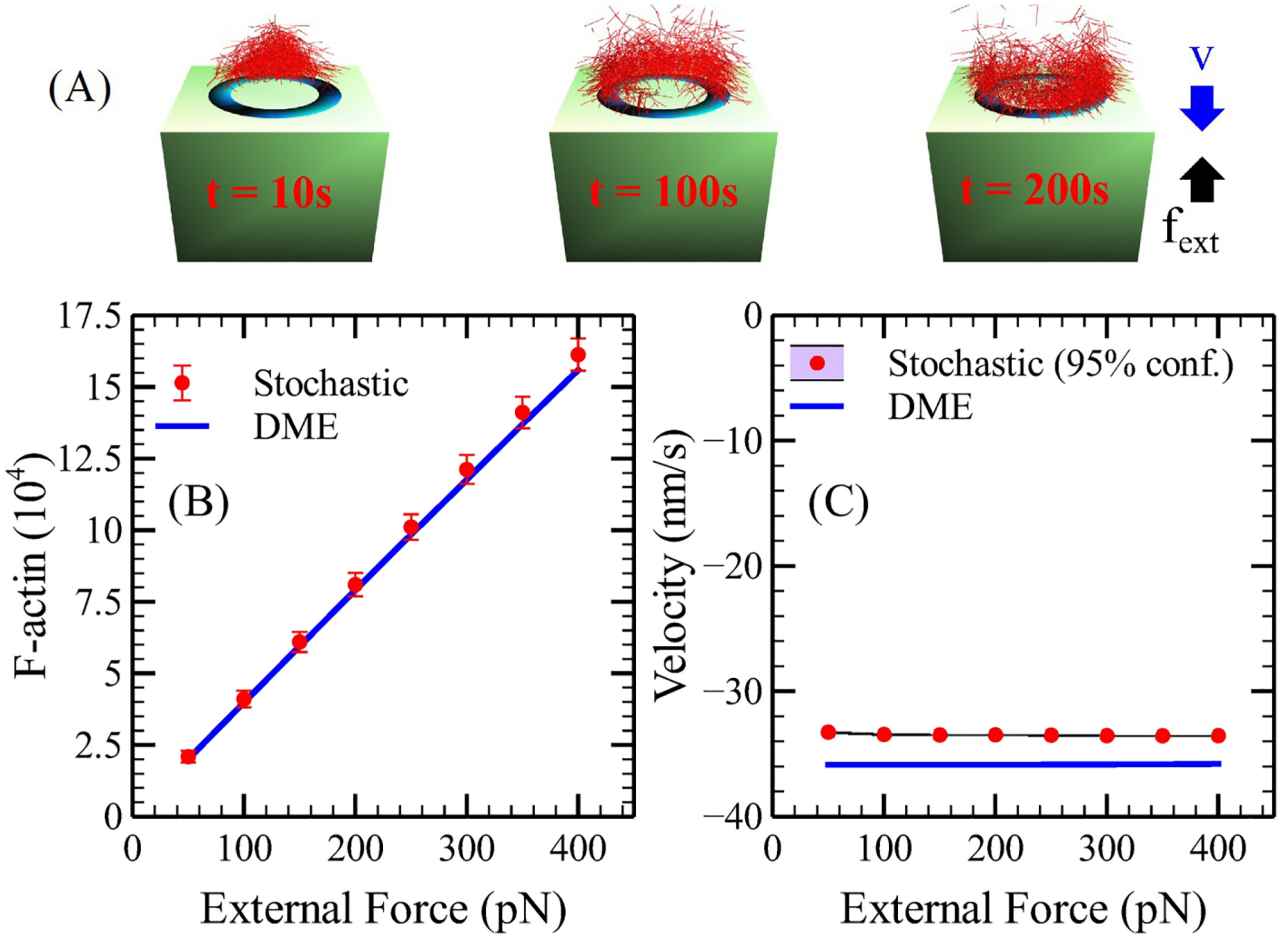
ME validation. We simulate (A) pushing of an obstacle by an actin network, using a stochastic method and the ME approach. We calculate (B) F-actin count and (C) velocity as functions of constant external force.

We assess the validity of the ME by examining how it treats a basic mechanochemical problem: an actin network pushing an obstacle that exerts a constant force opposing polymerization. The obstacle is coated with a ring-like NPF region, mimicking the Las17 ring in Ref. [12]; similar results are obtained for other NPF distributions, including a rectangle and a complete circle. In order to focus on the treatment of branched actin network growth, we ignore negative-feedback effects [12] of actin onto the NPFs. We compare ME results with stochastic simulation results. We start the calculation with 400 filaments, each of which has 50 subunits. For each force value, we run calculations for 200 seconds, and for the stochastic case run an ensemble of 100 simulation runs. The results, shown in Fig 3, show that the ME method agrees quantitatively with the stochastic simulations. We find that F-actin count depends linearly on the external force, and that the velocity is independent on the external force. Both findings are also consistent with the previous stochastic study in Ref. [32].

As mentioned above, key approximation of the ME is that filaments are assumed to grow instantaneously to their final lengths *l*(*y*, *t*), controlled by capping and force. This approximation is valid if the time scale of capping is much shorter than the characteristic time over which the actin count varies in the process of interest. The capping rate is on the order of 1*s*^−1^ in budding yeast [33], so the approximation should hold reasonably well for processes occurring on time scales of several seconds or more. This holds for the endocytosis system and model studied in Ref. [12], since the time scale of invagination is on the order of ten seconds. In addition, in our previous study, the assumption of instantaneous polymerization/capping gave results very similar to those of the explicit-polymerization methods when the same parameters were used, as shown by the “Four Variable” model in the Supplemental Material of [12]. Thus the range of validity of the ME approach includes the endocytosis model studied below, but otherwise will vary from case to case.

### Application to a mechanochemical model of endocytosis—Integrating actin dynamics with the cell membrane

We apply the ME method to CME by solving the actin network dynamics described by Eq (2), while simultaneously calculating membrane shape dynamics using the analysis of Ref. [5]. First, many possible mechanical equilibria corresponding to a range of force values are obtained as in Ref. [5]. These equilibria are given as a shape function for each value of the force. Then, the actin polymerization dynamics are calculated using the ME according to the shape function, and the process is repeated.

We focus on branching induced by the NPF Las17, which is the strongest one in budding yeast. Thus *N* = Las17 count. Recent superresolution data indicate that the NPF Las17 accumulates in a ring-shaped region, while the protein Sla2, which links F-actin to the cell membrane, accumulates at a spot inside the ring [34]. Therefore we divide the membrane into a ring of pushing forces corresponding to Las17 and a spot of pulling force corresponding to Sla2. The pushing forces are generated by the growth of branched filaments in the network. The pulling forces act on spontaneously nucleated filaments, which are assumed to form a passive layer attached to both Sla2 and the branched network. Sla2, with the membrane attached to it, is thus pulled back with the retrograde flow of the network (see Fig 1), as suggested in Ref. [11].

**Fig 4:**
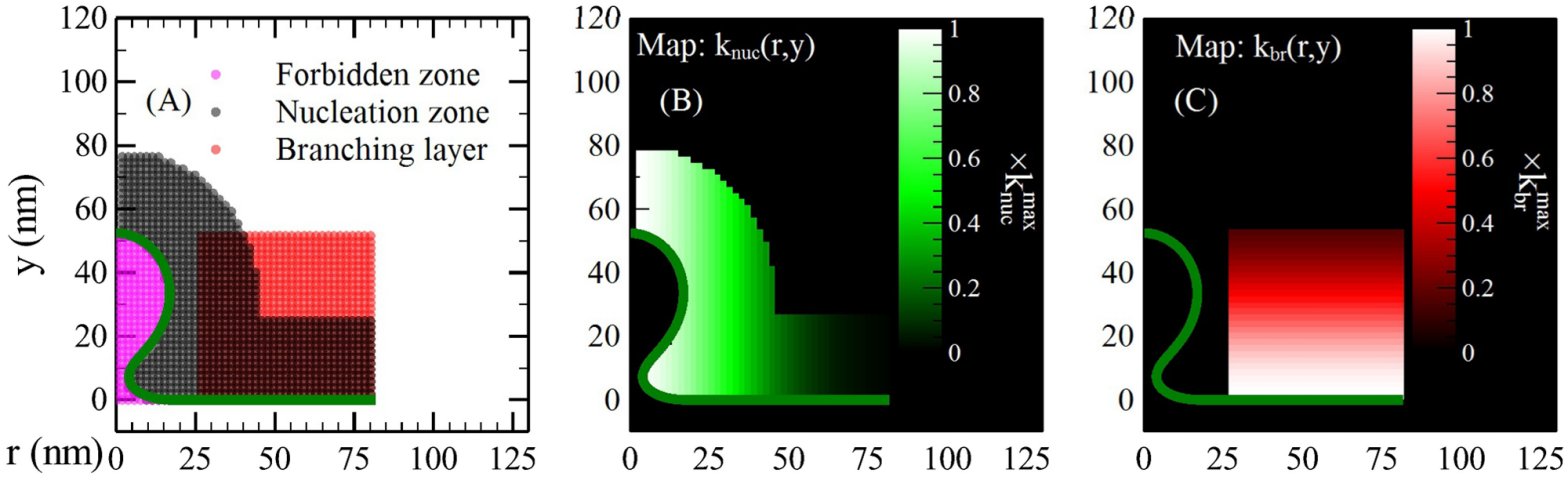
Spatial distribution of branching and spontaneous nucleation. (A) Branching and spontaneous-nucleation layers, and the forbidden zone, defined as the space inside the membrane where the filaments cannot penetrate. (B) The Gaussian nucleation function, for *k*_*nuc*_(*r*, *y*), Eq. (S33), defined in the black layer in (A). (C) The Gaussian branching function for *k*_*br*_(*r*, *y*), Eq (3), defined in the red layer in (A).

Actin polymerization also occurs mainly near the membrane [35]. We thus use the following form for the branching rate constant:

$$k_{br}\left(r,y\right)=\{\begin{array}{cc} k_{br}^{max}\text{exp}\left[-\frac{{\left(y-y_{L}\right)}^{2}}{2\sigma_{br}^{2}}\right] & \text{if}\;y_{L}<y<y_{L}+y_{br}\;\text{and}\;r_{L}^{in}<r<r_{L}^{out}\\ 0 & \text{otherwise} \end{array}$$ (3)

where $k_{br}^{max}$ is the maximum value of *k*_*br*_, *σ*_*br*_ is the width of the branching region (a precise definition is given in the Supplemental Material), *y*_*br*_ is a cutoff imposed for numerical convenience, and $r_{L}^{in}$ and $r_{L}^{out}$ are the inner and outer radii of the Las17 ring. Eq. (S33) gives the corresponding formula for *k*_*nuc*_. In practice, we use dimensionless rates ${\bar{k}}_{br}^{max}$ and ${\bar{k}}_{nuc}^{max}$, as in Eqs. S4 and S5. The spatial branching and nucleation functions are shown in Fig 4. Note that even though *k*_*br*_ cuts off sharply at *y* = *y*_*L*_, the dynamics of Eq 2 will result in a small component of *ρ* penetrating past *y*_*L*_. This portion of the *ρ* is used to calculate the pushing force of the actin onto the membrane below.

In our previous work [12], we demonstrated a crucial negative-feedback effect of actin branching on the Las17 count, and here we treat the Las17 dynamics using a similar rate equation:

$$\begin{array}{c} \frac{dN\left(t\right)}{dt}=N{\left(t\right)}^{2}\left[N_{full}-N\left(t\right)\right]-\alpha NF_{br}\left(t\right), \end{array}$$ (4)

where *N*_*full*_ is the maximum possible count of Las17 (from 2-d packing considerations), *α* is the probability that a branching event will cause Las17 to dissociate from the membrane (thus being inactivated), and

$$\begin{array}{c} F_{br}(t)=2\pi \int_{y_{L}}^{y_{L}+y_{br}}dy\int_{r_{L}^{in}}^{r_{L}^{out}}(\frac{a}{\sqrt{2}})k_{br}(r,y)\rho (r,y,t)rdr, \end{array}$$ (5)

is the number of new branches created per unit time per Las17 molecule. The probability *α* reflects the strength of binding of the Las17 to the membrane, and *a* = 2.7 *nm* is the step size per added subunit. The factor of $a/\sqrt{2}$ is the projection of *a* onto the *y*-axis.

**Fig 5:**
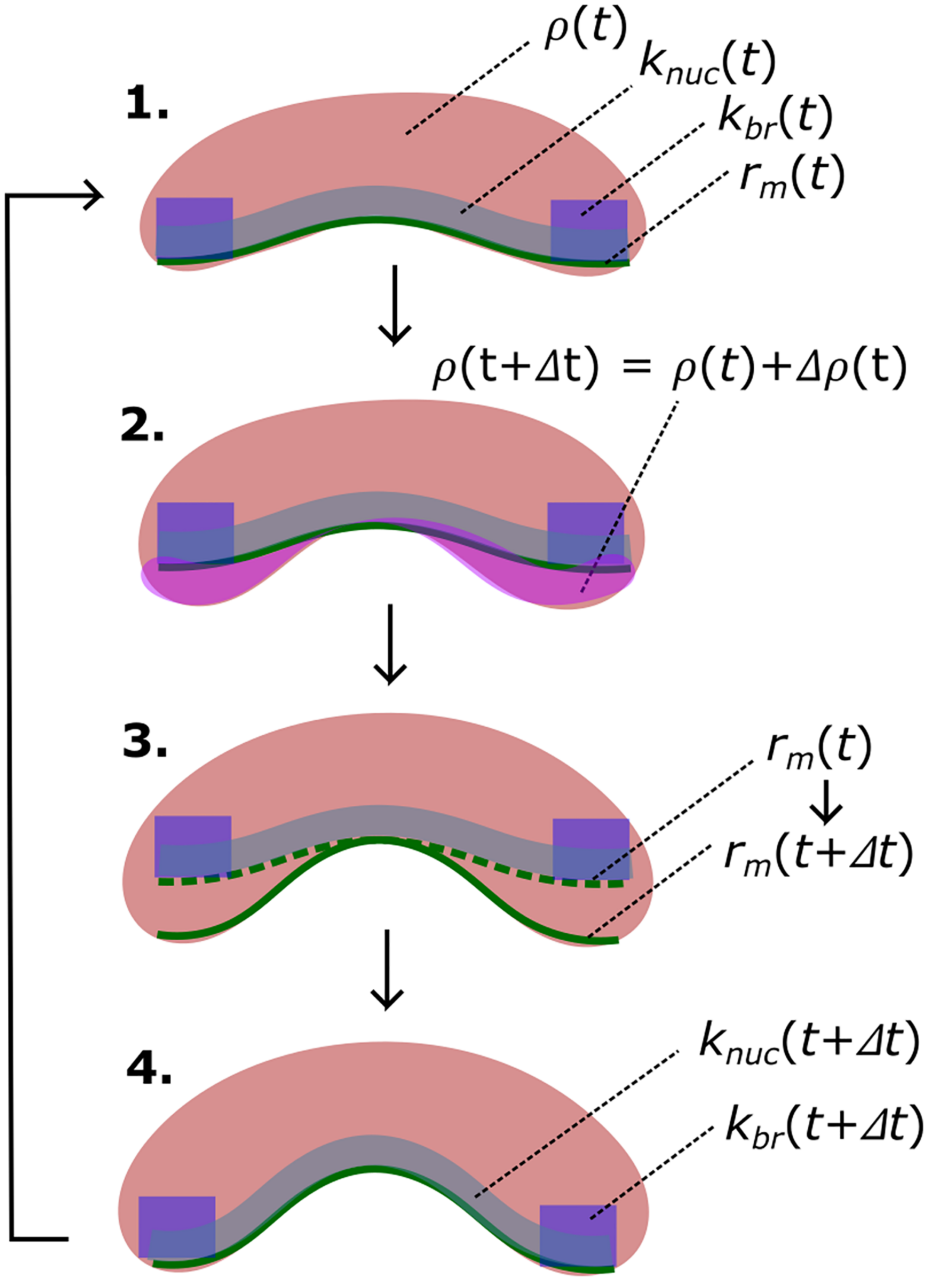
Computational flow. The four functions *ρ*, *k*_*nuc*_, *k*_*br*_ and *r*_*m*_ have initial values at time *t*. Then *ρ* is updated according to Eq. S5. Next *r*_*m*_ is updated according Eq. S31, using force balance (Eq. S26). Finally *k*_*nuc*_ and *k*_*br*_ are updated according to Eqs. S33 and 3. The process is repeated at each time step.

The forces from actin polymerization deform the membrane from one mechanical equilibrium to another, as indicated in Fig 5. The use of equilibrium shape functions is justified, because the kinetics of the actin-membrane system are determined by the slowly varying actin network shape (timescale > 1s), but relaxation of the membrane occurs much faster, at the speed of sound (timescale ∼ 0.001s).

Because the net force on the actin network is exceedingly small [4], the pushing force from the actin network must balance the pulling force, as shown in S2 Fig. The pushing force is generated by network growth in the outer region comprising the Las17 ring, and is thus denoted *f*_*out*_. It is calculated by allowing the actin network to protrude slightly into the membrane, according to Eq 2, and imposing a linear repulsive force between this portion of the actin and the cell membrane (see Supplemental Material for details). The pulling force is exerted in the inner region corresponding to the Sla2 spot, and is thus denoted *f*_*in*_. The balancing forces *f*_*in*_ and *f*_*out*_ produce a deformation described by the membrane shape function *y*_*m*_[*r*_*m*_(*s*)], where *y*_*m*_ is membrane height and *r*_*m*_ is the radial coordinate in the membrane.

During each time step, actin first polymerizes according to Eq 2. The membrane deformation, or invagination depth *y*_*I*_ is then determined from the extent of actin polymerization by a procedure implementing a “molecular clutch” based on the amount of F-actin. The possibility of such a mechanism is supported by findings [36] that a clutch transmits forces from the actin cytoskeleton to the extracellular matrix or other cells. A clutch should also be present in CME because there must be a transition in mechanical behavior with increasing F-actin count. When the F-actin count is small, there is very little actin material in the central region of the endocytic patch. Therefore there is almost nothing for the outer filaments, which are moving backwards in retrograde flow, to “grab” onto. This makes it impossible for the growing network to exert a pulling force. On the other hand, when the F-actin count is larger, there is enough material at the center to transmit the force generated by the growing filament to the endocytic coat proteins We implement the clutch as follows. Up to a certain minimum value of the F-actin count, *F*_*min*_, *y*_*I*_ is taken to vanish; for larger values of *F*, the actin network is assumed to be completely rigid, and *y*_*I*_ is driven by the difference in polymerization rates between the outside and the inside. Details are given in the Supporting Information.

Given *y*_*I*_, the deformation profile is chosen from the pretabulated set (see S3 Fig) as the one with invagination $y_{I}^{pre}$ closest to *y*_*I*_. The force is chosen by linear interpolation between the force of the profile with $y_{I}^{pre}$ and that of another profile with $y_{I}^{pre}\pm 0.1R_{\Pi }$, so that $y_{I}^{pre}-0.1R_{\Pi }<y_{I}<y_{I}^{pre}+0.1R_{\Pi }$. The updated shape function and force are used in the next step to determine the branching region, spontaneous-nucleation region and dynamics of actin polymerization (see Fig 5). This approach should describe the dynamics of the membrane deformation well, since the differences between successive membrane shape functions are relatively small.

The initial actin distribution is a ring of filaments represented by $\rho \left(r,y,0\right)=\left(1/2\pi r\right)\delta \left(r-r_{L}^{in}\right)\theta \left(y-y_{L}\right)\theta \left(y_{L}+l_{max}-y\right)$, where $r_{L}^{in}$ is the inner limit of the Las17 ring. Further, at the beginning of the simulation *L*(0) = 20, *y*_*S*_ = *y*_*L*_ = 0, and all forces vanish.

## Results

Here we describe our procedure for fitting the model to measured properties of endocytosis in budding yeast. Then we present several experimentally testable predictions: First, a spontaneous nucleation mechanism with a specific spatial location is required for adequate force generation. A substantial fraction of the nucleated filaments must be near the middle of network in order to exert sufficient pulling force to overcome the turgor pressure, being dragged along by the rest of the network as indicated in Fig 1. Second, we quantitatively predict the response of actin and NPF assembly to the drugs CK-666 and Latrunculin A (LatA), which suggests a new direction for quantitative experiments. Third, we constrain the values of key parameters. In our previous model [12], we found that changes in some key parameters can be compensated by changes in other parameters. For instance, a broad range of values of the polymerization rate gave results consistent with experiments; a lower polymerization rate could be compensated by higher branching rate, and vice versa. However, after including the membrane more completely in the new model, we find such compensation to be less effective, limiting the range of parameter values. A polymerization rate within a narrow range is required to sufficiently deform the membrane into “*Ω*” shapes and to correctly obtain the effects of NPF mutations.

### Model fitting

We use the experimentally measured time courses of F-actin (*F*) and Las17 number (*N*) [12] as our fitting targets. We use the four quantities (*k*_0_, ${\bar{k}}_{br}^{max}$, *k*_*sev*_, and *α*) (see Table 1) as our fitting parameters, and regard the rest of parameters as “fixed” (see Table 1 and S1 Table). The values of the fitting parameters are obtained by minimizing the mean-square difference between the measured *F* and *N* time courses on one hand, and the model and the experimental data on the other hand:

$$\begin{array}{c} \epsilon =\frac{1}{n_{N}}\sum_{i=1}^{n_{N}}\frac{{\left[N_{mod}\left(t_{i}\right)-N_{exp}\left(t_{i}\right)\right]}^{2}}{{\left[max\left(N_{exp}\right)\right]}^{2}}+\frac{1}{n_{F}}\sum_{i=1}^{n_{F}}\frac{{\left[F_{mod}\left(t_{i}\right)-F_{exp}\left(t_{i}\right)\right]}^{2}}{{\left[max\left(F_{exp}\right)\right]}^{2}}, \end{array}$$ (6)

while keeping the “fixed” parameters unchanged. Here *n*_*N*_ and *n*_*F*_ are the number of experimental data points of Las17 and F-actin in the time courses. *F*_*exp*_ is obtained from measurements of the time course of Abp1, as in Ref. [12]. At each step of the the fitting process, we randomly vary (*k*_0_, ${\bar{k}}_{br}^{max}$, *k_sev_*, *α*) and calculate *ϵ*. The new values are accepted if *ϵ* is lower. The above computation is repeated until *ϵ* does not decrease despite a large number of attempts (typically about 300). Then the values of the fitting parameters are found for a given set of “fixed” parameters. This process requires a large number of trial calculations (about 300) for one set of the “fixed” parameters, which is nevertheless manageable within the ME method. In the stochastic simulations, one needs to repeat the calculation about 1000 times for the same fitting parameter values to obtain adequate statistics. Thus, a total in the range of 300,000 runs are needed for one set of “fixed” parameters, which is a very demanding computational load. In Ref. [12], we estimated the fitting parameters by first pre-fitting a simplified four-variable rate-equation model to the experimental time courses, and then fitting the stochastic model to experimental maxima and lifetimes starting with the pre-fitted parameter values. This process is less efficient and accurate than the automatic fitting process used here, which is difficult to incorporate into the stochastic model.

**Table 1:** Core parameter values in the default model. Note that the branching and nucleation rates are given as scaled parameters, defined in the Supplementary Material.

Fitting parameters		Fixed parameters		
parameter/units	value	parameter/units	value	estimation method
*k*_0_ (10^−5^ *s*^−1^)	7.24			
${\bar{k}}_{br}^{max}$ (10^−3^ *s*^−1^)	2.59	${\bar{k}}_{nuc}^{max}$ (10^−3^ *s*^−1^)	15	constrained by pulling force and invagination depth
*k*_*sev*_ (*s*^−1^)	0.36	${\bar{l}}_{max}$	60	5.3 *μM* actin concentration [33]
*α*	0.082			

Other parameters are listed in S1 Table.

There are two additional “fixed” parameters that are estimated via either other experimental data or physical constraints (see Table 1). The zero-force dimensionless filament length ${\bar{l}}_{max}$ (the maximal number of subunits) in Table 1 is estimated as ${\bar{l}}_{max}=k_{on}G/k_{cap}$, where *k*_*on*_ = 11.6*μM*^−1^ *s*^−1^ [37] is the on-rate constant, *G* = 5.3*μM* [33] is the free-actin concentration, and the capping rate *k*_*cap*_ is taken to be 1 *s*^−1^ [33]. The spontaneous nucleation rate parameter $k_{nuc}^{max}$ is fixed by a combination of two constraints: i) that adequate pulling forces can be generated, and ii) that sufficient invagination can be obtained.

Additional parameters, including those describing the geometry, are given in S1 Table.

### Wild-type results

**Fig 6:**
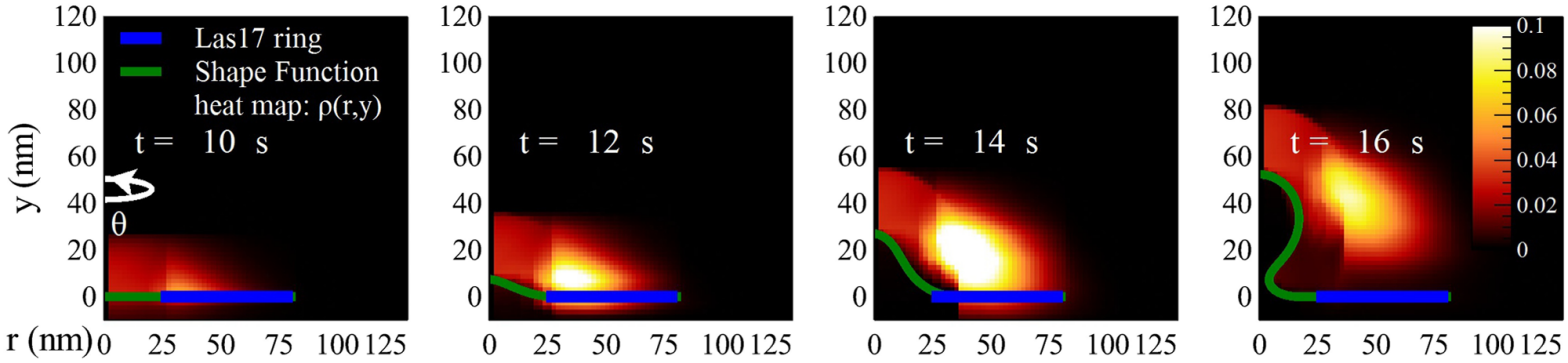
Time evolution of the F-actin distribution and the membrane profile. The F-actin density *ρ*(*r*, *y*) (red) at four time points during the time course of endocytosis in wild-type cells is shown by the heat map. The cell membrane is in green and the Las17 is in blue.

**Fig 7:**
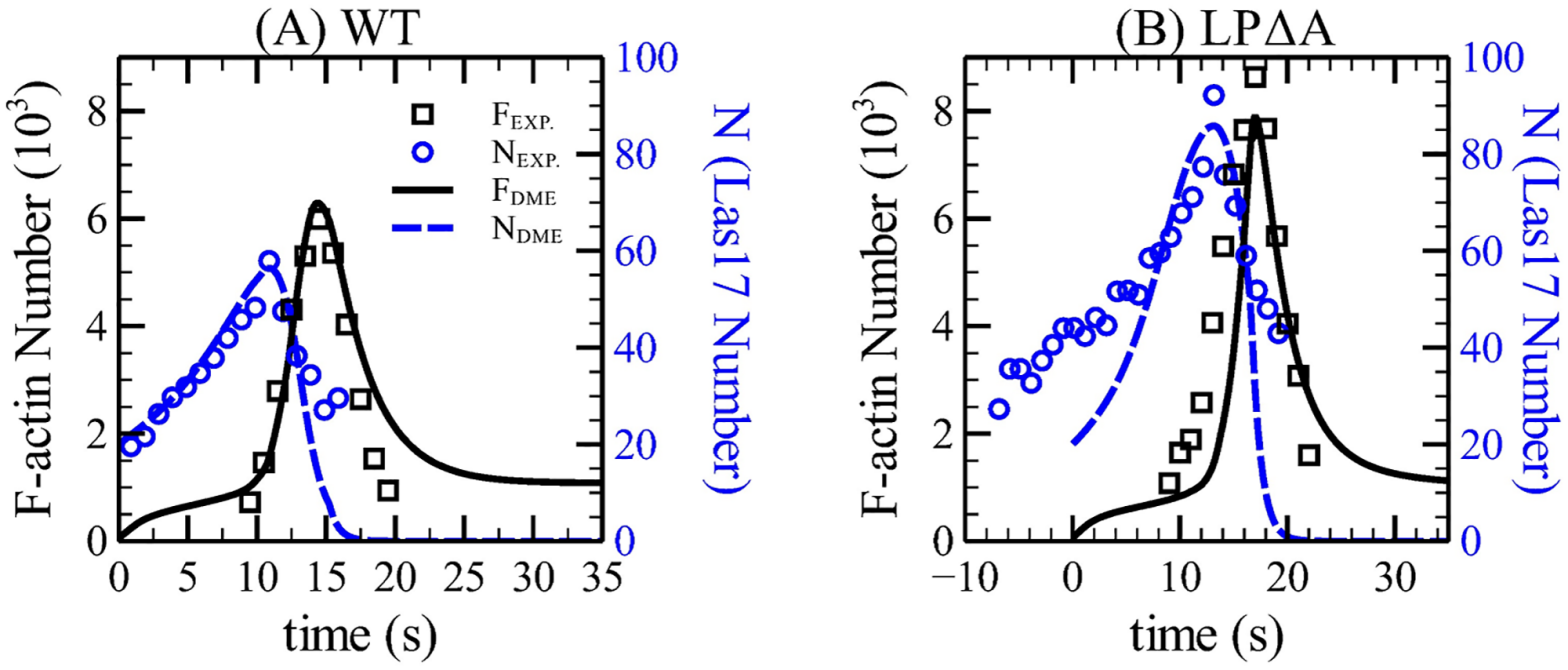
Time courses of F-actin (*F*) and Las17 (*N*) of wild-type and *las17 pan1Δacidic* (LPΔA) cells. (A) Time courses of *F* and *N* obtained from the ME model, compared to experimental data [12] for wild-type cells. (B) Same comparison for LPΔA cells, in which ${\bar{k}}_{br}^{max}$ is reduced by 40%.

Fig 6 shows how the actin network invaginates the membrane over time. The membrane forms an ‘*Ω*’ shaped invagination after 16 seconds into the simulation or about 5 seconds after actin polymerization starts, consistent with observations in electron micrographs [15]. The time courses of the F-actin count *F* and Las17 count *N* also reveal a good fit to the experimental data in Ref [12], shown in Fig 7A. Note that the actin distribution extends slightly below the plane of the membrane, and outside the Las17 ring. This occurs because of the nonlocal dynamics of Eq 2. The spreading outside the Las17 ring is physically expected because of the nonzero filament length. The portion below the membrane plane is an approximation used to calculate the pushing force generated by the actin, as described in the Supplemental Material.

The three-dimensional distributions predicted here could be tested by superresolution microscopy methods with resolution on the scale of tens of nanometers. Such methods [34] have found that F-actin forms a hemispherical shape, and Las17 forms a ring, as in the present model. Electron microscopy data in the literature [15, 38, 39] are also consistent with an F-actin hemisphere.

### Mutation of active region of Las17

The “acidic” regions of Las17 and other yeast NPFs are believed to control their binding to Arp2/3 complex, and therefore their NPF activity. The mutant containing mutations of both the Las17 and the NPF Pan1, *las17 pan1Δacidic* (abbreviated as LPΔA) should have a strong reduction in the Las17 branching activity. We choose this mutant to avoid possible compensatory effects from the nucleation-promoting activity of Pan1. As in Ref. [12], we model the mutation via a 40% reduction in $k_{br}^{max}$. Fig 7B shows that the model matches the measured LPΔA phenotype [7] well, with an accuracy comparable to that of our previous model [12]. Remarkably, the F-actin count is actually increased by reducing *k*_*br*_. This counter-intuitive phenotype results from a competition between a direct effect and an indirect effect. The direct effect is the reduction in branching rate per molecule of Las17 caused by the mutation. The indirect effect is the resulting increase in Las17 caused by the reduced branching rate, due to the negative-feedback effect described in Eq (4). This increase will tend to increase the F-actin count. For the conditions considered here, the indirect effect outweighs the direct one.

### A spontaneous actin nucleation mechanism is required for adequate pulling forces

**Fig 8:**
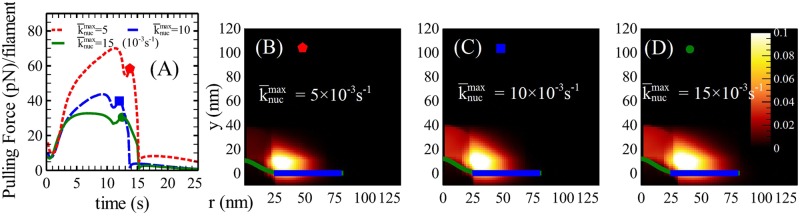
Time evolution of the pulling force per filament for different spontaneous nucleation rates, with representative *ρ*(*r*, *y*) plots. (A) Time courses of the pulling force per filament for ${\bar{k}}_{nuc}^{max}=$ 33%, 66% and 100% of the default value (Table 1). (B)-(D) Representative *ρ*(*r*, *y*) for each case; (D) represents the default model. The representative time point is chosen when the pulling force per filament reaches its maximum value while the invagination is greater than zero. The actin network in the middle is significantly sparser in (B) than in (D), causing the pulling force per filament to exceed the rupture force measured in Ref. [42].

As indicated in Fig 1, actin filaments in the central region are required to exert pulling forces. We assume that these filaments arise from spontaneous nucleation (not requiring NPFs at the endocytic site). Possible sources of the spontaneously nucleated actin filaments can be severed filament fragments [40], or nucleation via Dip1, which is independent of NPFs [41]. A minimum value of ${\bar{k}}_{nuc}^{max}$ is required to exert adequate pulling force, since reducing ${\bar{k}}_{nuc}^{max}$ reduces the number of filaments in the central region (see Fig 8B–8D). This increases the pulling force per filament, and eventually causes them to detach from the membrane.

The maximum pulling force that a membrane-attached filament can sustain is not known. But Ref. [42] gives a quantitative measurement (> 40*pN*) of the rupture force between a single actin filament and the crosslinking proteins filamin and *α*-actinin *in vitro*, at low loading rates. In red blood cells [43], the interaction force between the actin cytoskeleton and the membrane was found to be ∼10*pN* per filament in a model fitted to experimental data. However the cytoskeleton-membrane interactions in yeast could be very different from those in red blood cells. We thus based our estimate of the maximum actin filament pulling force on the measured rupture force. To estimate the pulling force per filament in our model, we divided the total maximum pulling force of 725 pN obtained from the membrane-energetics analysis (Supplementary Material) by the number of pulling filaments, estimated as the total F-actin count inside $r_{L}^{in}$ divided by $\sqrt{2}y_{nuc}/a$, a dimensionless measure of the height of the nucleation layer. In the default model, this procedure gave a pulling force of ∼30*pN* per filament, below the rupture force [42]. When spontaneous nucleation was suppressed by reducing ${\bar{k}}_{nuc}^{max}$, the pulling force exceeded the rupture force, as shown in Fig 8A. Thus a minimum rate of spontaneous filament nucleation in the central region is required for the actin network to pull the membrane without rupture of the actin-membrane interactions.

This prediction might be tested by deletion of the protein Dip1, which could participate in an NPF-independent actin polymerization pathway [41]. If Dip1 nucleates filaments in the central region, its deletion should have two main effects. First, the actin hemisphere should be sparser in the middle. as shown in Fig 8B. This could be verified by superresolution images of *dip1*Δ cells. Second, in the *dip1*Δ cells, reduced nucleation should lower the number of pulling filaments (see Fig 8A), causing rupture if the force per filament exceeds the rupture force. This would reduce the efficiency of invagination. Ref. [41] found that in *dip1*Δ cells, only 40% of the total patches were internalized, compared to ∼90% in WT cells. Internalization of 30% of the patches was also delayed delayed by over 20 seconds. Both of these effects could be due to the reduced number of filaments in the central region.

On the other hand, we find that too large a magnitude of ${\bar{k}}_{nuc}^{max}$ also disrupts invagination. We increased ${\bar{k}}_{nuc}^{max}$ by a factor of 2 and refitted. In comparison with the default invagination $y_{l}^{max}=54$ nm, we obtained a maximum invagination $y_{l}^{max}=30$ nm, which we consider to be a failed event. Therefore, possible values of ${\bar{k}}_{nuc}^{max}$ are in a range limited by the constraints of i) adequate pulling force and ii) adequate invagination.

### Partial inhibition of branching and polymerization cause different characteristic responses

We investigate the responses of the actin-membrane system to the drugs CK-666, which inhibits branching, and Latrunculin A (LatA), which inhibits polymerization and to some extent branching. S4 Fig summarizes the effects on *F*_*max*_ and $y_{I}^{max}$ of a broad range of combinations of parameter changes. Note than in S4 Fig frame D, breaking of pulling filaments bearing very large loads is not included. Inclusion of this effect will lead to failure of invagination at small ${\bar{k}}_{nuc}^{max}$, as discussed above.

#### CK-666 treatment

**Fig 9:**
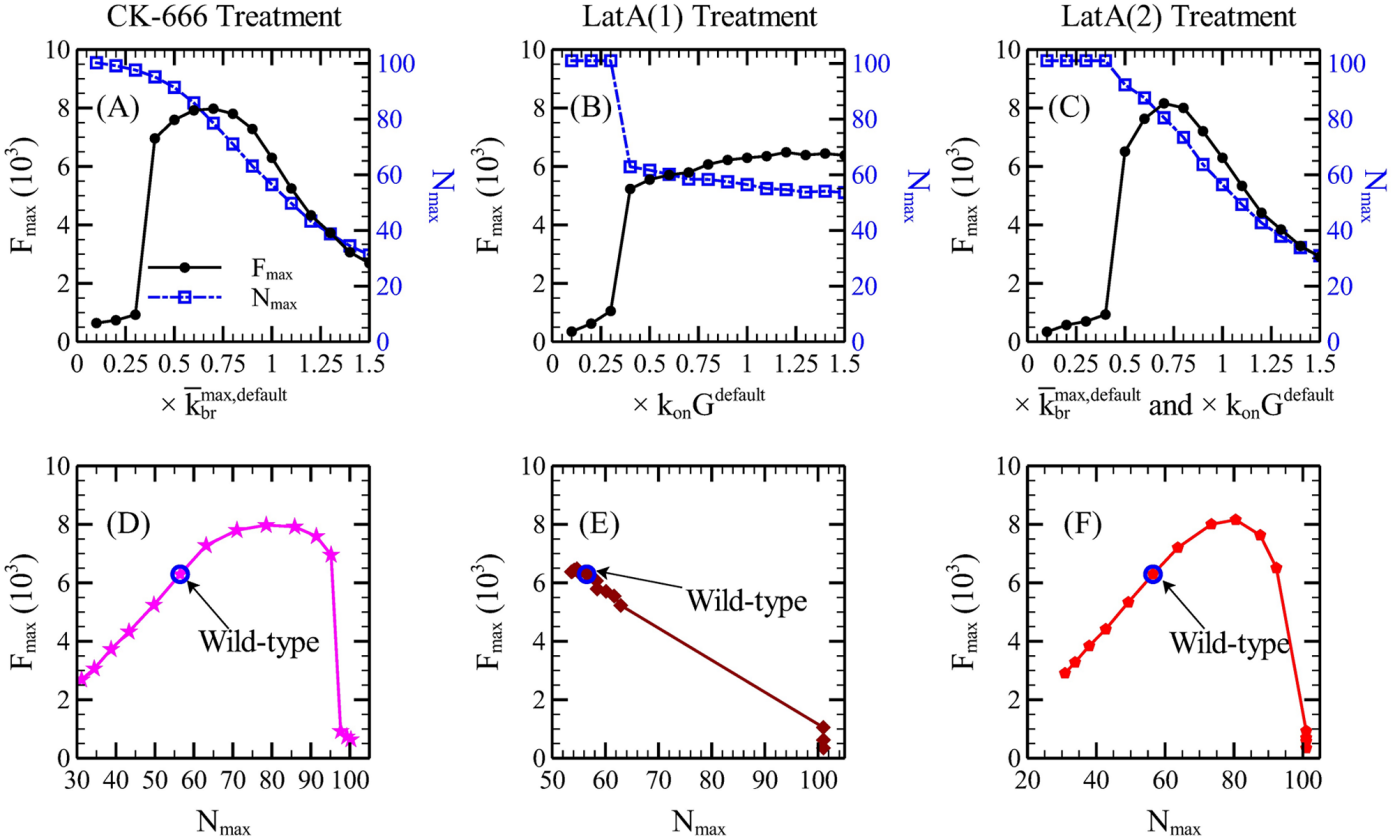
Responses of *F*_*max*_ and *N*_*max*_ to treatment with the drugs CK-666 and LatA, and the corresponding phase diagrams. We assume that (A) CK-666 reduces ${\bar{k}}_{br}^{max}$, (B) LatA(1) reduces *k*_*on*_ *G* only, and (C) LatA(2) reduces both ${\bar{k}}_{br}^{max}$ and *k*_*on*_ *G*. The *N*_*max*_−*F*_*max*_ plots in frames (D)-(F) can be directly compared to experiments. The wild-type data points are denoted by blue open circles, and decreasing ${\bar{k}}_{br}^{max}$ and/or *k*_*on*_ *G* corresponds to moving right in these frames.

Fig 9A shows the effect on *F*_*max*_ and *N*_*max*_ of gradually reducing ${\bar{k}}_{br}^{max}$, to mimic CK-666 treatment. *N* monotonically increases as ${\bar{k}}_{br}^{max}$ is reduced, due to the negative feedback term in Eq 4. *F* increases first and then dramatically decreases. This non-monotonic behavior, like the behavior for the LPΔA mutant discussed above, results from the negative feedback effect of branching onto *N*. The reduction in branching per Las17 molecule increases *N* because the effect of the negative feedback from F-actin is reduced. The total extent of branching, depending on the product of *N* and ${\bar{k}}_{br}^{max}$, also increases, until ${\bar{k}}_{br}^{max}$ is decreased by about 60%, at which point it begins to decrease. This explains the increase in *F*_*max*_. These results cannot be directly compared to experiments, because the extent of reduction of ${\bar{k}}_{br}^{max}$ corresponding to a given concentration of CK-666 is not known. For this reason, we replot our results in the *N*_*max*_-*F*_*max*_ plane, where both of the axes can be experimentally measured. Fig 9D shows such a plot, where ${\bar{k}}_{br}^{max}$ decreases to the right going from the default value indicated with a circle. With decreasing ${\bar{k}}_{br}^{max}$, *F*_*max*_ and *N*_*max*_ first increase together. Then the curve flattens out, at *F*_*max*_ ∼ 8000. With further reduction in ${\bar{k}}_{br}^{max}$, *F*_*max*_ drops sharply over a small range of *N*_*max*_. The non-monotonic asymmetric bell shape plot is directly testable by experiments.

We also find that the maximum invagination length $y_{I}^{max}$ is slightly increased down to the critical value of ${\bar{k}}_{br}^{max}$ (see S4 Fig frame C and D). We see no way of measuring this effect directly, but it suggests that the efficiency of endocytosis should not be impaired by small doses of CK-666.

#### LatA treatment

LatA sequesters free monomers and thereby reduces the polymerization rate *k*_*on*_ *G*, where *k*_*on*_ is the on-rate constant and *G* is the actin concentration. It may also reduce *k*_*br*_, but it is not known by how much. We thus treat two different assumptions regarding its action, i) that only the polymerization rate is increased, and ii) that the branching rate and polymerization rate are reduced by the same amount.

We take a reduction of only the polymerization rate into account by reducing ${\bar{l}}_{max}$, according to the relationship ${\bar{l}}_{max}=k_{on}G/k_{cap}$ obtained for a filament polymerizing over a time 1/*k*_*cap*_. We plot our results in terms of the fractional reduction in *k*_*on*_ *G*, which equals the fractional reduction in ${\bar{l}}_{max}$. Fig 9B shows that there is no increase in *F*_*max*_ in this case. Rather, *F*_*max*_ decreases and *N*_*max*_ increases monotonically with decreasing *k*_*on*_ *G*. There is a sudden jump in both *F*_*max*_ and *N*_*max*_ where *k*_*on*_ *G* is reduced by 60%. Fig 9E shows the corresponding *N*_*max*_-*F*_*max*_ plot. The shape is much simpler than the one in Fig 9D. *F*_*max*_ decreases nearly linearly with *N*_*max*_.

We also found that $y_{I}^{max}$ is significantly reduced upon reducing *k*_*on*_ *G* (see Fig 4C), an effect opposite to that seen for reduction of *k*_*br*_.

We take simultaneous reduction of branching and polymerization into account by assuming that *k*_*on*_ *G* and ${\bar{k}}_{br}^{max}$ are reduced by the same percentage relative to the default values. According to Ref. [44], successful branching requires the binding between Arp2/3 and an actin monomer. Thus suggests a linear dependence of branching rate on the free-actin concentration, the same as for *k*_*on*_ *G*. We find that both the response curve and the *N*_*max*_-*F*_*max*_ plot (Fig 9C and 9F) are similar to those for the CK-666 treatment (Fig 9A and 9D). In this case it is thus clear that the effect of reduced branching dominates that of reduced polymerization. Additionally, the maximum invagination length $y_{I}^{max}$ is slightly increased as ${\bar{k}}_{br}^{max}$ and *k*_*on*_ *G* are jointly reduced, and decreases with further reduction (see S4 Fig frame C).

### A limited range of polymerization-rate values predict effective invagination and a correct mutant phenotype

#### Small values of the polymerization rate *k*_*on*_ *G* lead to shallow invagination, which can not be compensated by increasing branching

**Fig 10:**
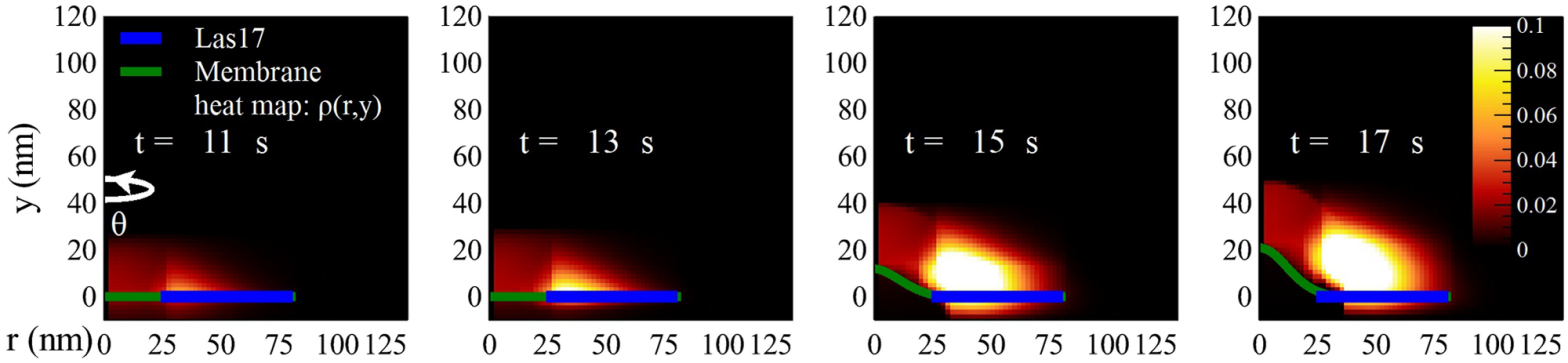
Time evolution of the F-actin distribution and the membrane profile for low polymerization rate. In this model, we have reduced ${\bar{l}}_{max}$ to 30, half its default value (see Table 1). This corresponds to a reduction of 50% in the polymerization rate. The actin network cannot effectively invaginate the membrane.

We reduced *k*_*on*_ *G* by 50% and then refit the four fitting parameters. Although the modified model matches the time courses of *F* and *N*, its actin network can not effectively invaginate the membrane, as seen in Fig 10. Only a shallow invagination is formed (20*nm* compared to 54*nm* from the default model), even though the refitted ${\bar{k}}_{br}^{max}=3.19\times 10^{-3}s^{-1}$ is significantly larger than the default ${\bar{k}}_{br}^{max}$ (see Table 1). Thus, we find that branching and polymerization are not functionally redundant.

This prediction could be tested by actin underexpression [45], or by capping protein overexpression [46], which would reduce ${\bar{l}}_{max}$ by increasing *k*_*cap*_. In either case, invagination should be reduced. Gradual treatment with Latrunculin A, as discussed above, would have some of the effects of reducing *k*_*on*_ *G*, but might also change $k_{br}^{max}$.

#### Large *k*_*on*_ *G* values lead to insensitivity to LPΔA mutation

On the other hand, increasing *k*_*on*_ *G* by 100% leads to a prediction for the LPΔA phenotype that is inconsistent with the one observed in Ref. [7]. The maximum values of *F* and *N* during their time courses, *F*_*max*_ and *N*_*max*_, initially increase when ${\bar{k}}_{br}^{max}$ is reduced. However, *F*_*max*_ does not increase as much as was experimentally observed [12]. Thus, we find that polymerization faster than our default value reduces the sensitivity of the actin network to NPF mutations.

Therefore, a range of *k*_*on*_ *G* values near the value in Table 1 is required by the constraints of proper invagination length and LPΔA phenotype. This was not found by our previous model [12], which had no explicit membrane treatment.

## Discussion

### Computational efficiency of ME

The ME enables a “one-shot” approach to stochastic actin network dynamics by treating the pdf.

It represents a statistical average of many ensembles of the same actin network calculated by the equivalent stochastic simulation, which is referred as “*the infinite population limit*” in Ref. [47]. We feel that the use of a statistical average is legitimate, because endocytosis in yeast is highly stereotypical. The behavior of the actin network typically displays a single mode value [7] (the most likely value) plus a range of fluctuations, instead of having multiple mode values.

The “one-shot” nature of the ME simplifies the process of making modifications and refitting the model to experimental data. For both ME and stochastic methods, calculation of branching processes causes the largest computational load. The ME approach is most efficient when the branching region is small because it scans the physical space for possible branching events. The stochastic simulation, on the other hand, is most efficient when the total number of filaments is small, because it scans the subunits for possible branching sites. Thus, the ME is computationally most powerful for treating dense actin networks with relatively small regions of NPF, especially with spatial symmetry.

### Application range of ME approach

The ME approach could be applied to many other problems involving branched network growth, as long as the branching is generated by a flat NPF region at a membrane or surface. For cell migration, the cytoskeleton can sometimes be simplified to a two dimensional network [48] or even a one dimensional network [49] with NPFs close to the cell membrane. The ME method is well suited to this type of problem. Notice that because the ME method is mechanochemical, it could be used to describe mechanical feedback effects on cell migration, if combined with a “Helfrich”-type calculation of the cell membrane forces. The ME method is also well-suited for treating filopodium and lamellipodium geometries where the membrane forces can be calculated straightforwardly from the membrane geometry.

### Limits of ME approach

In order to develop a practical method for treating a problem as complex as endocytosis, we have made several simplifying assumptions.

#### Simplified treatment of mechanics

The actin network is treated as infinitely rigid when *F* > *F*_*min*_, so deformations arising from stresses, such as cytoskeletal tension, acting on semiflexible filaments, are not treated. Although the absence of an actin cortex in yeast precludes cortical tension, tension and compression are both present inside the endocytic actin patch. Despite the semiflexible nature of the actin filaments, assuming infinite rigidity may be reasonable on the basis of our previous estimate [50] of the Young’s modulus of the actin patch in the range of 140 kPa to 500 kPa. These high values result from the large density of crosslinkers in the patch.

However, the response of the network to stress, resulting from filament semiflexibility, could be added to the method by using a coordinate transformation. In our current approach of imposing a 45 degree angle in the actin network, the expression for the spatial coordinates in terms of the lattice coordinates has a cos(*ϕ*) factor, where *ϕ* = 45°. Semiflexibility could be included by allowing *ϕ* to be force dependent. Under network compression, *ϕ* would increase. The actin network would thus be squeezed by the force. Moderate levels of squeezing could to a positive feedback between actin growth and force, in two ways. First the actin density will be higher in the branching layer close to the membrane, thus increasing branching. Second, because the cosine factor decreases under opposing force, polymerization will be enhanced. On the other hand, if the network becomes soft enough, invagination will fail [4].

#### Simplified treatment of actin turnover. Actin turnover is known to affect force generation by cytoskeletal networks

It is treated in a simplified fashion here, using a first-order decay term with rate *k*_*sev*_. In reality, turnover is probably a higher-order process involving hydrolysis and binding of accessory proteins such as cofilin. However, regardless of the nature of the process, the main effect on CME within the present physical picture is the following: Too high a rate of turnover will reduce the F-actin count below the level required to sustain invagination. Too low a rate would result in excessive polymerization, which would reduce the available G-actin concentration either by global depletion or local depletion at the endocytic site by diffusion limitations. We have assumed that the turnover rate in wild-type cells is between these limits, allowing effective invagination.

#### Absence of myosin-based contractility from model

Type II myosins, which in combination with actin generate contractility, are absent at the endocytic patch. The Type I myosins present at the patch bind only one actin filament (plus presumably the membrane), and it is not known whether they can generate contractile forces in yeast. However, Type I myosin motor activity is important for endocytosis in yeast [51]. Furthermore, *in vitro* experiments [52] have demonstrated that Type I myosins can tubulate giant unilamellar vesicles (GUVs), against small opposing forces, by pulling out on the membrane while moving along preexisting actin filaments. It is not known whether such a mechanism could function against the larger force barrier caused by the turgor pressure in yeast.

The ME method will lose its validity under conditions including i) small system size, ii) low values of the F-actin density, iii) large opposing force per filament, and iv) slow capping. If any of i)—iii) occur, a fully stochastic calculation will produce bimodal or multimodal behavior (which in the case of endocytosis could include a fraction of patches that fail to internalize), while the ME method will incorrectly predict a single type of behavior. If the capping is slow relative to polymerization, then the extent of branching may be overestimated because filaments can extend unrealistically fast into the branching region.

### Conclusion

We have developed a computationally efficient master equation (ME) approach for calculating the spatial distribution of F-actin branched networks growing in the presence of mechanical forces. The approach was validated by comparison with stochastic-simulation results. It was then used to develop a mechanochemical model of clathrin-mediated endocytosis in yeast (CME), which treats both the actin network and the cell membrane realistically. The mechanochemical model was used to reveal the time evolution of the actin-membrane system during CME, to quantitatively estimate unknown parameter values, and to predict several important mechanisms in CME that are unseen or omitted in previous models. These predictions provide possible directions for experiments in CME, especially for superresolution microscopy and drug treatments. Beyond CME, the new ME approach provides possible applications to a wide range of problems involving the spatial distribution of branched actin polymerization.

## Supporting information

S1 Supporting Information Describes details of computational method and choice of parameter values. (PDF)

S1 FigIllustration of the three dimensional ME, for the case $\bar{l}=4$.(TIF)

S2 Fig Illustration of the spatial and mechanical variables. (TIF)

S3 FigExamples of pretabulated shapes.We calculate 80 shapes with heights of $y_{I}^{pre}=0.1R_{\Pi },0.2R_{\Pi }...8.0R_{\Pi }$, using the method of Ref. [5]. (A) Shallow shapes. (B) *Ω* (deeply invaginated) shapes. (C) Membrane force *f*_*in*_ at each height $y_{I}^{pre}$.(TIF)

S4 FigVariation of *F*_*max*_ and $y_{I}^{max}$ under combinations of parameter changes.(A) *F*_*max*_ vs. ${\bar{k}}_{br}^{max}$ and *k*_*on*_ *G*^*default*^. (B) *F*_*max*_ vs. ${\bar{k}}_{br}^{max}$ and ${\bar{k}}_{nuc}^{max}$. (C) $y_{I}^{max}$ vs. ${\bar{k}}_{br}^{max}$ and *k*_*on*_ *G*^*default*^. (D) $y_{I}^{max}$ vs. ${\bar{k}}_{br}^{max}$ and ${\bar{k}}_{nuc}^{max}$. Effects of breaking of pulling filaments occurring at small ${\bar{k}}_{nuc}^{max}$ are not included.(TIF)

S1 Table Complete set of parameters and functions, and their physical interpretation. (PDF)
