# Supplementary material for: A master equation approach to actin polymerization applied to endocytosis in yeast
Source: PLoS Comput Biol. 2017 Dec 14;13(12):e1005901. doi: 10.1371/journal.pcbi.1005901 (PMC5746272; doi:10.1371/journal.pcbi.1005901)
Supplement: S1 Supporting Information — (PDF) [file pcbi.1005901.s001.pdf]

A Master Equation Approach to Actin Polymerization Applied to Endocytosis in Yeast

X. Wang and A. E. Carlsson

Supplementary Material

1. Discretization of θ. We approximate the integral in Eq. 2 by choosing a $n_\theta = 8$ mesh for each height y to calculate the θ-integral:

$$\frac{\partial \rho(r,y,t)}{\partial t} \approx \frac{1}{n_\theta}\int_0^{l(y,t)} dy' k_{br}(r, y+y') N(t) \sum_{i=0}^{n_\theta} \rho\left[R(r,\theta_i), y+y', t\right] - k_{sev}\rho(r,y,t) + k_{nuc}(r,y,t), \quad \text{(S1)}$$

where

$$R(r,\theta_i) = \sqrt{y'^2 + r^2 - 2ry' cos(\theta_i)}, \quad \text{(S2)}$$

is the radius of the ith subunit at each given height that branches through (r, y, t), and

$$\theta_i = \frac{2\pi}{n_\theta} i, \quad \text{(S3)}$$

is the ith mesh angle. To check that n_θ is large enough, we performed some calculations with $n_\theta = 16$, which gave changes of much less than 1% in the F-actin count.

2. Discretization of r and t. We set the time step as $\Delta t = 0.005s$. We use $\Delta r = a/\sqrt{2}$ as the smallest spatial increment because of the approximately $\pm 45°$ orientation of the filaments with respect to y. In two dimensions, Eq. 1, corresponding to 2, becomes

$$\frac{\Delta\bar{\rho}(\bar{x},\bar{y},t)}{\Delta t} \approx \sum_{\bar{y}'=0}^{\bar{l}(\bar{y},t)} \frac{\bar{k}_{br}(\bar{x}, \bar{y}+\bar{y}')}{2} N(t)\left[\bar{\rho}(\bar{x}-\bar{x}', \bar{y}+\bar{y}', t) + \bar{\rho}(\bar{x}+\bar{x}', \bar{y}+\bar{y}', t)\right] - k_{sev}\bar{\rho}(\bar{x},\bar{y},t) + \bar{k}_{nuc}(\bar{x},\bar{y},t), \quad \text{(S4)}$$

where scaled variables are denoted by bars. For example, $\bar{\rho}$ is the dimensionless F-actin distribution. The dimensionless length variables are $\bar{x} = x/\Delta r$, $\bar{x'} = x'/\Delta r$, $\bar{y} = y/\Delta r$ and $\bar{y}' = y'/\Delta r$,$\bar{y} = y/\Delta r$ and $\bar{y}' = y'/\Delta r$; the scaled rates are $\bar{k}_{br} = k_{br}\Delta r$ and $\bar{k}_{nuc} = k_{nuc}\Delta r^2$; $\bar{l}(\bar{y}, t) = l(y,t)/\Delta r$ is the filament length (an integer), see Eq. S30, which takes the value 4 in 2..

In three dimensions, Eq. S1 becomes

$$\frac{\Delta\bar{\rho}(\bar{r},\bar{y},t)}{\Delta t} \approx \frac{1}{n_\theta}\sum_{\bar{y}'=0}^{\bar{l}(\bar{y},t)} \bar{k}_{br}(\bar{r}, \bar{y}+\bar{y}') N(t) \sum_{i=0}^{n_\theta} \bar{\rho}\left[R(\bar{r},\theta_i), \bar{y}+\bar{y}', t\right] - k_{sev}\bar{\rho}(\bar{r},\bar{y},t) + \bar{k}_{nuc}(\bar{r},\bar{y},t), \quad \text{(S5)}$$

where $\bar{r} = r/\Delta r$, and now $\bar{k}_{nuc} = k_{nuc}\Delta r^3$.

3. Rule for updating y-positions of outer Las17 ring and central Sla2 cap. The positions of the Las17 and Sla2 regions on the membrane are determined by the

mechanical equilibrium between the actin force and the membrane force. We use the following algorithm to find this equilibrium,

$$\frac{dy_L(t)}{dt} = \frac{1}{\gamma} f_{net}(t), \tag{S6}$$

where $y_L(t)$ is the position of the Las17 ring at t, $f_{net}(t)$ is the net force on the Las17 ring, consisting of the contribution from the actin network and the membrane force, with the latter obtained from the membrane model in Ref. [1].

The time evolution of the Sla2 cap y-position depends on whether or not there is enough F-actin present to bend the membrane by exerting pulling forces. We assume that the pulling force "kicks in" when the F-actin count reaches a minimum value F_{min}. The y-positions y_S and y_L of the Sla2 cap and the Las17 ring are taken relative to the actin network. When $F < F_{min}$, the Sla2 cap moves with the Las17 ring, and there is no bending. When $F > F_{min}$, the Sla2 cap stays put relative to the actin network. Thus

$$y_S(t) = \begin{cases} y_L(t) & \text{when } F < F_{min} \\ y_S(t-\Delta t) & \text{otherwise,} \end{cases} \tag{S7}$$

where Δt is the time step. Initially the actin network pushes the Las17 ring to move along the y direction, and the Sla2 cap follows because there is no force to bend the membrane. Later, when $F > F_{min}$, the Sla2 cap is anchored to the stationary actin network and thus remains fixed, but the Las17 ring is still pushed by the actin network to move in the y direction. A separation between the Sla1 and Las17 is then created. The F-actin may thus be considered to be a molecular clutch for creating bending by moving the Sla2 cap relative to the Las17 ring.

4. Calculation of membrane deformation profile from invagination length y_I. Given $y_I(t) = y_L(t) - y_S(t)$, we calculate the deformation profile and the corresponding force using the method of Ref. [1]. First, we solve the Euler-Lagrange equations [1], based on the general analysis of Ref. [2].

   The equations are made dimensionless as in Ref. [1]. We use a turgor pressure value $\Pi = 0.2\ MPa$ recently obtained [3] by a high-speed indentation method that avoids some potential pitfalls of slower methods. Then a spatial scale $R_\Pi = 15nm$ corresponding to the observed invagination shapes is used to determine a bending modulus $\kappa = 2\Pi R_\Pi^3 = 328 k_B T$, which is the unit of energy. This value is comparable to the value of $285 \pm 30 k_B T$ measured for clathrin-coated vesicles [4]. The corresponding force scale is $f_\Pi = 2\Pi\kappa/R_\Pi = 565pN$. The turgor pressure $\Pi = 0.2MPa$. Then the pressure scale is 2Π, so the dimensionless pressure is $\bar{\Pi} = 0.5$. The dimensionless spontaneous curvature, as in Ref. [1], is $\bar{C}_0 = C_0 R_\Pi = 0.4$. We take the membrane tension to be $\sigma = 0$ because it is mechanically insignificant [1]. In the actin network calculations, R_Π is multiplied by a factor $(\sqrt{2}/a)$ to make it compatible with the actin coordinates, giving a final value of $\bar{R}_\Pi = 7.85$.

   Then the Euler-Lagrange equations are:

$$\ddot{\psi} = \frac{\cos\psi}{r_m}\left(\frac{\sin\psi}{r_m} - \dot{\psi} + \eta - f_{in}\right) + \frac{\bar{\Pi}}{2} r_m \cos\psi + \frac{\nu}{r_m}\sin\psi, \quad \text{(S8)}$$

$$\dot{\nu} = \frac{1}{2}\left(\dot{\psi} - \bar{C_0}\right)^2 - \frac{\sin^2\psi}{2r_m^2} + \bar{\sigma} + \bar{\Pi} r_m \sin\psi, \quad \text{(S9)}$$

$$\dot{\eta} = 0, \quad \text{(S10)}$$

$$\dot{r_m} = \cos\psi, \quad \text{(S11)}$$

$$\dot{y_m} = -\sin\psi, \quad \text{(S12)}$$

where the geometrical variables are illustrated in Fig. S2, while ν and η are Lagrange multipliers. Here f_{in} is a point force that acts at the peak of the invagination, but which affects the Lagrange equations of motion at all values of s. A "shooting" method is used to solve them, starting with six boundary conditions at an outer boundary chosen where the membrane is flat (right side in Fig. S2), where we define $s = 0$:

$$\psi\,(s=0) = 0, \quad \text{(S13)}$$

$$\dot{\psi}\,(s=0) = -\sqrt{\bar{C_0}^2 + 2\sigma} \quad \text{(S14)}$$

$$\nu\,(s=0) = 0 \quad \text{(S15)}$$

$$\eta = 0, \text{ constant}, \quad \text{(S16)}$$

$$r_m\,(s=0) = r_m^i \text{ (a free parameter)}, \quad \text{(S17)}$$

$$y_m\,(s=0) = 0. \quad \text{(S18)}$$

We have taken $\eta = 0$, which holds when the z-coordinate of one of the endpoints is not fixed (see Ref. [2]). The boundary conditions for $\dot{\psi}$ and ν are taken from the Supplemental Material of Ref. [1]. Then, for a given value of y_I, r_m^i and f_{in}, as well as the total arc length s_{tot} from $s = 0$ to the top of the invagination, are varied, until Eqs. (S8-S12) satisfy the following boundary conditions at the highest point in Fig S2:

$$r_m(s = s_{tot}) = 0, \quad \text{(S19)}$$

$$y_m(s = s_{tot}) = y_I, \quad \text{(S20)}$$

$$\psi(s = s_{tot}) = 0. \quad \text{(S21)}$$

The shape function is then defined from $s = 0$ to s_{tot} as

$$y_m\,(s) \equiv y_m\,[r_m\,(s)\,, y_I\,(t)]\,. \quad \text{(S22)}$$

Each shape function has a uniquely associated actin force value [1]. Thus, the inward pulling force required to bend the membrane to form the shape described by Eq. (S22) is defined as the membrane force function,

$$f_{in}\,(t) \equiv f_{in}\,[y_I\,(t)]\,. \quad \text{(S23)}$$

For computational convenience, we pre-calculated a set of shapes with discrete invagination lengths of $y_I^{pre} = 0.1R_\Pi$, $0.2R_\Pi ... 9R_\Pi$, where $R_\Pi = 15nm$ is the length scale. Thus we have a discrete set of forces values $f_{in}^{pre}\,[y_I^{pre}]$. We linearly interpolate between the f_{in}^{pre} values to obtain the continuous membrane force function $f_{in}\,[y_I\,(t)]$.

5. Molecular force clutch. To treat the initiation of deformation, we use a different approach to calculate the membrane force for $y_I < 0.1R_\Pi$. We assume, as above, that a minimum F-actin count F_{min} is required to transmit pulling forces through the actin network, and thus use a broadened transition of the following form:

$$f_{in}\left[0\right] = \frac{1}{2}\left\{\left[\tanh A(F - F_{min})\right] + 1\right\} f_{in}\left[0.1R_\Pi\right], \tag{S24}$$

where $A = 0.005$.

6. Mechanical equilibration procedure. We balance the pushing force on the Las17 region from the actin network, calculated using a linear approximation, with the pulling force f_{in} on the Sla2 region resulting from membrane bending.
   (a) Start with $y_S(t) = y_L(t) = 0$ (see Fig. S2).
   (b) Calculate $\rho(r, y, t)$ using 2. The forces enter $\rho(r, y, t)$ through the filament length as described in subsection 7 below.
   (c) Calculate $y_L(t)$ and $y_S(t)$ based on the force balance.
      i. Calculate the linear pushing force with a spring constant k_{hmc} exerted by the actin network based on the configuration of the F-actin below the Las17 ring as following

$$f_{out}\left(t\right) = k_{hmc}\int_{-\infty}^{y_L(t)} dy\left[y - y_L\left(t\right)\right]\int_0^{2\pi} d\theta \int_0^{\infty} rdr\rho\left(r, y, t\right). \tag{S25}$$

      ii. Update $y_L(t)$ using Eq S6.
      iii. Update $y_S(t)$ using Eq S7.
      iv. Repeat i), ii) and iii) until

$$f_{net}\left(t\right) = f_{in}\left[y_I\left(t\right)\right] + f_{out}\left(t\right) < 1pN. \tag{S26}$$

      To reduce the computational load, we choose a relatively large $1/\gamma$ so that f_{net} converges quickly. In some cases, this γ value is too large and f_{net} does not converge. In these cases we reduce $1/\gamma$ by 90% and keep repeating i), ii) and iii). If convergence still fails, then we scan over an interval of y_L to find the force-balancing y_L value.
   (d) Repeat b) and c) to calculate the actin network and the membrane force for the next time step.

7. Calculation of filament length. First, a Brownian ratchet factor [5] is defined as

$$B\left(t\right) = \exp\left[-\frac{|f_{out}\left(t\right)|a}{n_{att}\left(t\right)k_B T}\right], \tag{S27}$$

where $a = 2.7$ nm is the length of the actin monomer, k_B is Boltzmann's constant, T is the temperature, and

$$n_{att}\left(t\right) = \int_0^{2\pi} d\theta \int_0^{\infty} rdr\frac{1}{3}\left[\rho\left(r, y_L - a, t\right) + \rho\left(r, y, t\right) + \rho\left(r, y_L + a, t\right)\right] \tag{S28}$$

is the number of filaments attached to the membrane. This factor gives the expected reduction of the polymerization velocity due to the opposing force. The projected length of a branched filament terminating at y is taken to be

$$l\left(y, t\right) = \begin{cases} l_{max} & \text{when } y - y_L\left(t\right) \geq 0, \\ \left[y_L\left(t\right) - y\right]\left[1 - 1/B\left(t\right)\right] + l_{max} & \text{else.} \end{cases} \tag{S29}$$

In the first case the last subunit of the new branch is above the membrane, while in the second case the last subunit of the new branch impinges the membrane. Thus part of the new branch is affected by the Brownian ratchet factor, reducing the length. For example, the case of a filament growing into the membrane, with its base at y_L, corresponds to $y_L(t) - y = l(y,t)$. In this case one readily shows that $l(y,t) = l_{max}B$, as expected from the Brownian-ratchet analysis. After normalization by Δr and discretization,

$$\bar{l}(\bar{y},t) = \begin{cases} \bar{l}_{max} & \text{when } \bar{y} - \bar{y}_L(t) \geq 0, \\ \lfloor [\bar{y}_L(t) - \bar{y}][1 - 1/B(t)] + l_{max} \rfloor & \text{else.} \end{cases}, \quad \text{(S30)}$$

where $\lfloor x \rfloor = max\{n \in \mathbb{Z} | n \leqslant x\}$ is the floor function and $\bar{l}_{max} = l_{max}/(a/\sqrt{2})$ is the number of subunits in a filament grown in the absence of force.

8. Spatial distribution of spontaneous filament nucleation. First, we find r_m as the inverse function of Eq. S22:

$$r_m(s) \equiv r_m[y_m(s), y_I(t)]. \quad \text{(S31)}$$

Then, we define the spontaneous-nucleation layer as the set of points satisfying the following condition:

$$r_m[y_m(s), y_I(t)] < r(y) < r_m[y_m(s) - y_{nuc}, y_I(t)] + r_{nuc}. \quad \text{(S32)}$$

Thus the spontaneous-nucleation region extends from the membrane by a distance r_{nuc} in the r direction and a distance y_{nuc} in the y-direction, as shown in 4. Within the spontaneous-nucleation layer,

$$k_{nuc}(r,y,t) = k_{nuc}^{max} \exp\left(-\frac{r^2}{2\sigma_{nuc}^2}\right), \quad \text{(S33)}$$

where the values of y_{nuc}, r_{nuc}, and σ are given in Table S1. Also, branching is absent in the forbidden zone in 4, which is defined by

$$y(r) < y_m[r_m(s), y_I(t)]. \quad \text{(S34)}$$

9. Stochastic simulations for validation of the ME method. The procedure is the same as that described in the Supplemental Material of Ref. [6], except that we replace explicit polymerization with instantaneous branching using the length function in Eq. S29. The NPF ring in the validation system has an inner radius of $80a$ and an outer radius of $120a$, chosen to be in line with typical dimensions of systems that might be studied by the method, and the simulations contain enough subunits that the overall behavior (for example constant F-actin count vs. oscillations) can be straightforwardly determined. To enable the most direct comparison of the ME method with stochastic calculations, we do not include the negative-feedback term that is present in our application to endocytosis. In the absence of this term, smaller systems have large fluctuations that render the comparison of the ME with stochastic simulations difficult.

10. Choice of parameter values. The value of the parameters in Table S1 not referenced or discussed above are estimated as follows:

    The value $F_{min} = 500$, about 10% of F_{max}, is chosen to be large enough to avoid excessively large forces on individual filaments, and small enough that pulling and pushing forces are exerted over most of the polymerization time course.

For $k_{hmc} = 0.5pN/a$, we find that values larger than this cause the actin network to be pushed away from the membrane so much that the number of attached filaments is reduced, resulting in excessive forces per filament. If the value is too small, the actin network penetrates unphysically far into the membrane.

The value $1/\gamma = 1.0pN^{-1} \times a$ is a choice of computational convenience. Since it does not represent physical dynamics, we simply choose its value to quickly find the invagination length required for achieving equilibrium at each time step.

The parameters r_{nuc} and y_{nuc} are chosen to limit spontaneous nucleation to the spatial region near the membrane.

The parameter σ_{br} is chosen so that the branching region, after being modified by the Gaussian function, provides a number of branching spots similar to that in Ref. [6].

The decay parameter σ_{nuc} is chosen so that spontaneous nucleation is mainly confined to the center of the protein patch.

The sources or justifications for the remaining parameter values are given in Table S1.

References

1. Dmitrieff S, Nédélec F. Membrane mechanics of endocytosis in cells with turgor. PLoS Comput Biol. 2015;11(10):e1004538.

2. Jülicher F, Seifert U. Shape equations for axisymmetric vesicles: a clarification. Physical Review E. 1994;49(5):4728.

3. Goldenbogen B, Giese W, Hemmen M, Uhlendorf J, Herrmann A, Klipp E. Dynamics of cell wall elasticity pattern shapes the cell during yeast mating morphogenesis. Open Biology. 2016;6(9):160136.

4. Jin AJ, Prasad K, Smith PD, Lafer EM, Nossal R. Measuring the elasticity of clathrin-coated vesicles via atomic force microscopy. Biophysical journal. 2006;90(9):3333–3344.

5. Peskin CS, Odell GM, Oster GF. Cellular motions and thermal fluctuations: the Brownian ratchet. Biophysical journal. 1993;65(1):316–324.

6. Wang X, Galletta BJ, Cooper JA, Carlsson AE. Actin-regulator feedback interactions during endocytosis. Biophysical journal. 2016;110(6):1430–1443.

7. Dominguez R, Holmes KC. Actin structure and function. Annual review of biophysics. 2011;40:169.

Table S1. Complete set of parameters and functions, and their physical interpretation.

Parameter/Function	Meaning / Source or Estimation Method	Value
k_0 (parameter)	Autocatalytic assembly rate of Las17 / fitting	See Table 1
$k_{br}(r, y)$ (function)	Branching rate of the F-actin filaments / main text	-
$l(y)$ (function)	Length of newly branched F-actin filaments / main text	-
k_{sev} (parameter)	Severing rate of the F-actin filaments / fitting	Table 1
α (parameter)	Detachment rate of Las17 caused by branching / fitting	Table 1
$k_{nuc}(r, y)$ (function)	Nucleation rate / main text	-
N_{full} (parameter)	Maximum number of Las17 molecules / [6]	100
F_{min} (parameter)	F-actin threshold / Supplementary Material 10	500
a (parameter)	F-actin polymerization step size / [7]	2.7nm
k_{hmc} (parameter)	Spring constant in the actin force f_{out} / Supplementary Material 10	$0.5pN/a$
$1/\gamma$ (parameter)	Friction constant / Supplementary Material 10	$1.0pN^{-1} \times a$
r_{nuc} (parameter)	Limit of spontaneous nucleation layer / Supplementary Material 10	$10a$
y_{nuc} (parameter)	Limit of spontaneous nucleation layer / Supplementary Material 10	$10a$
y_{br} (parameter)	Limit of branching layer / [6]	$20a$
r_L^{in} (parameter)	Inner radius of Las17 ring / [6]	$10a$
r_L^{out} (parameter)	Outer radius of Las17 ring / [6]	$30a$
σ_{nuc} (parameter)	Gaussian width in $k_{nuc}(r, y)$ / Supplementary Material 10	r_L^{in}
σ_{br} (parameter)	Gaussian height in $k_{br}(r, y)$ / Supplementary Material 10	$0.5y_{br}$
κ (parameter)	Bending modulus of membrane / [1]	$328k_BT$
Π (parameter)	Turgor pressure / [3]	$0.2MPa$
R_Π (parameter)	Length scale / [1]	$15nm$
C_0 (parameter)	Spontaneous curvature / [1]	$0.4/R_\Pi$
σ (parameter)	Membrane tension / [1]	0

Fig S1. Illustration of 2, the three dimensional ME, for the case $\bar{l} = 4$.

Fig S2. Illustration of the spatial and mechanical variables. Note that the upper case S indicates the Sla2 cap, and the lower case s is the coordinate variable along the membrane.

Fig S3. Examples of pretabulated shapes. We calculate 80 shapes with heights of $y_I^{pre} = 0.1R_\Pi, 0.2R_\Pi ... 8.0R_\Pi$, using the method of Ref. [1]. (A) Shallow shapes. (B) Ω (deeply invaginated) shapes. (C) Membrane force f_{in} at each height y_I^{pre}.

Fig S4. Variation of F_{max} and y_I^{max} under combinations of parameter changes. (A) F_{max} vs. $\bar{k}_{br}^{max}$ and $k_{on}G^{default}$. (B) F_{max} vs. $\bar{k}_{br}^{max}$ and $\bar{k}_{nuc}^{max}$. (C) y_I^{max} vs. $\bar{k}_{br}^{max}$ and $k_{on}G^{default}$. (D) y_I^{max} vs. $\bar{k}_{br}^{max}$ and $\bar{k}_{nuc}^{max}$. Effects of breaking of pulling filaments occurring at small $\bar{k}_{nuc}^{max}$ are not included.
