# Supplementary figures and images for: A master equation approach to actin polymerization applied to endocytosis in yeast
Source: PLoS Comput Biol. 2017 Dec 14;13(12):e1005901. doi: 10.1371/journal.pcbi.1005901 (PMC5746272; doi:10.1371/journal.pcbi.1005901)

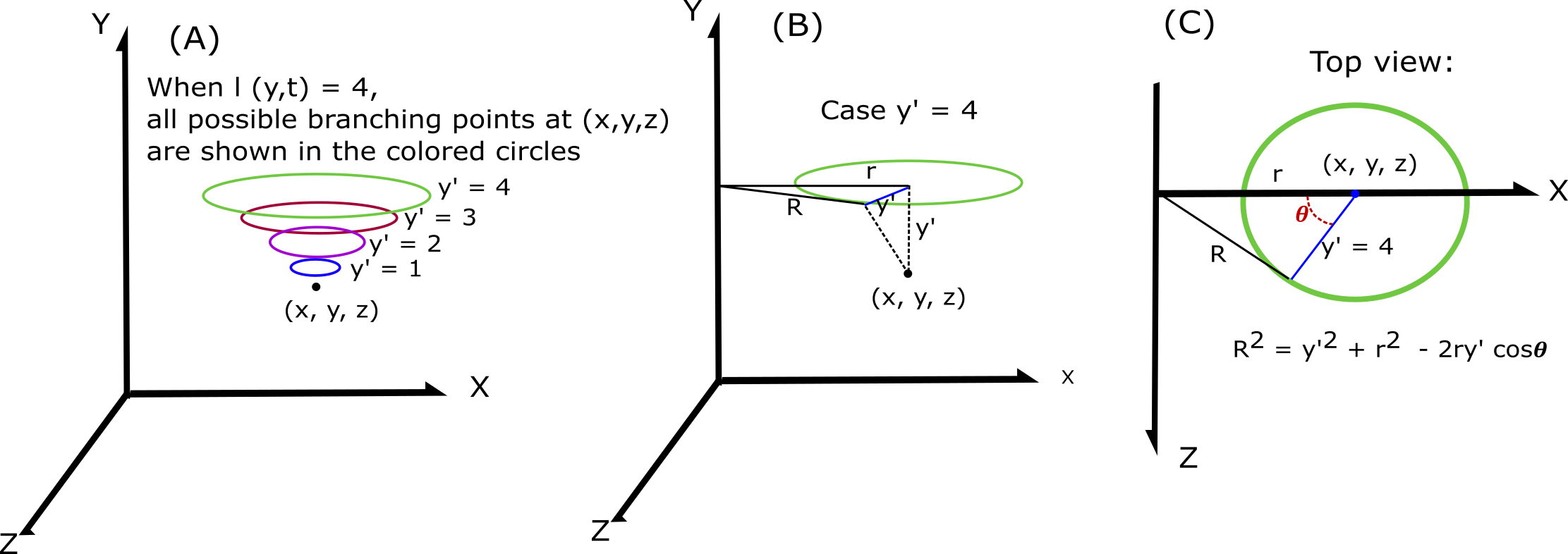

Supplement: S1 Fig — (TIF) [file pcbi.1005901.s002.tif]

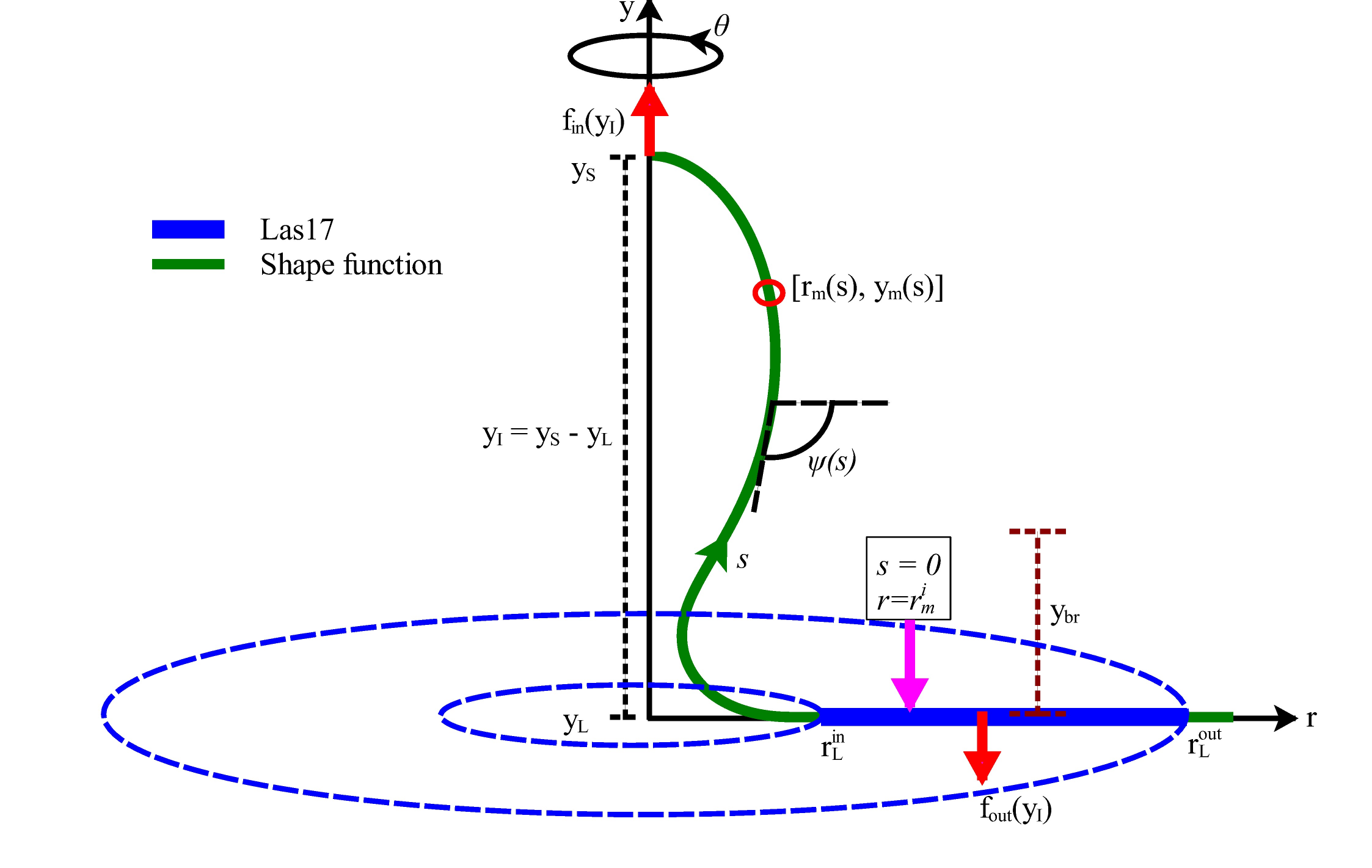

Supplement: S2 Fig — (TIF) [file pcbi.1005901.s003.tif]

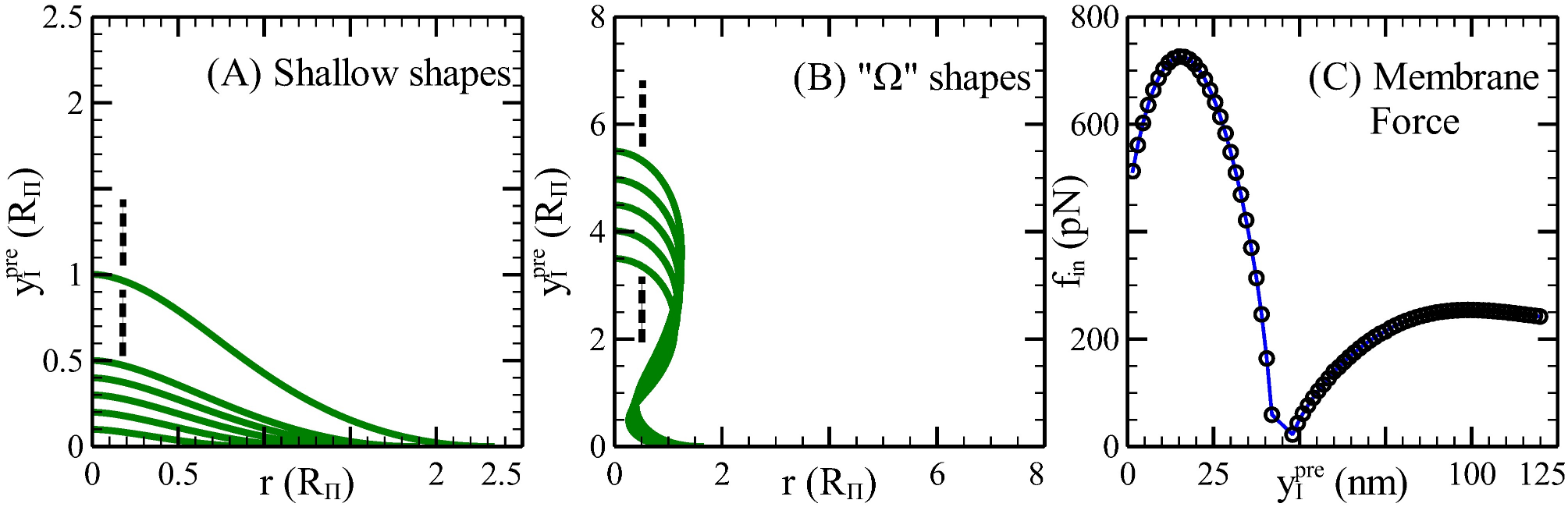

Supplement: S3 Fig — We calculate 80 shapes with heights of yIpre=0.1RΠ,0.2RΠ...8.0RΠ, using the method of Ref. [5]. (A) Shallow shapes. (B) Ω (deeply invaginated) shapes. (C) Membrane force fin at each height yIpre. (TIF) [file pcbi.1005901.s004.tif]

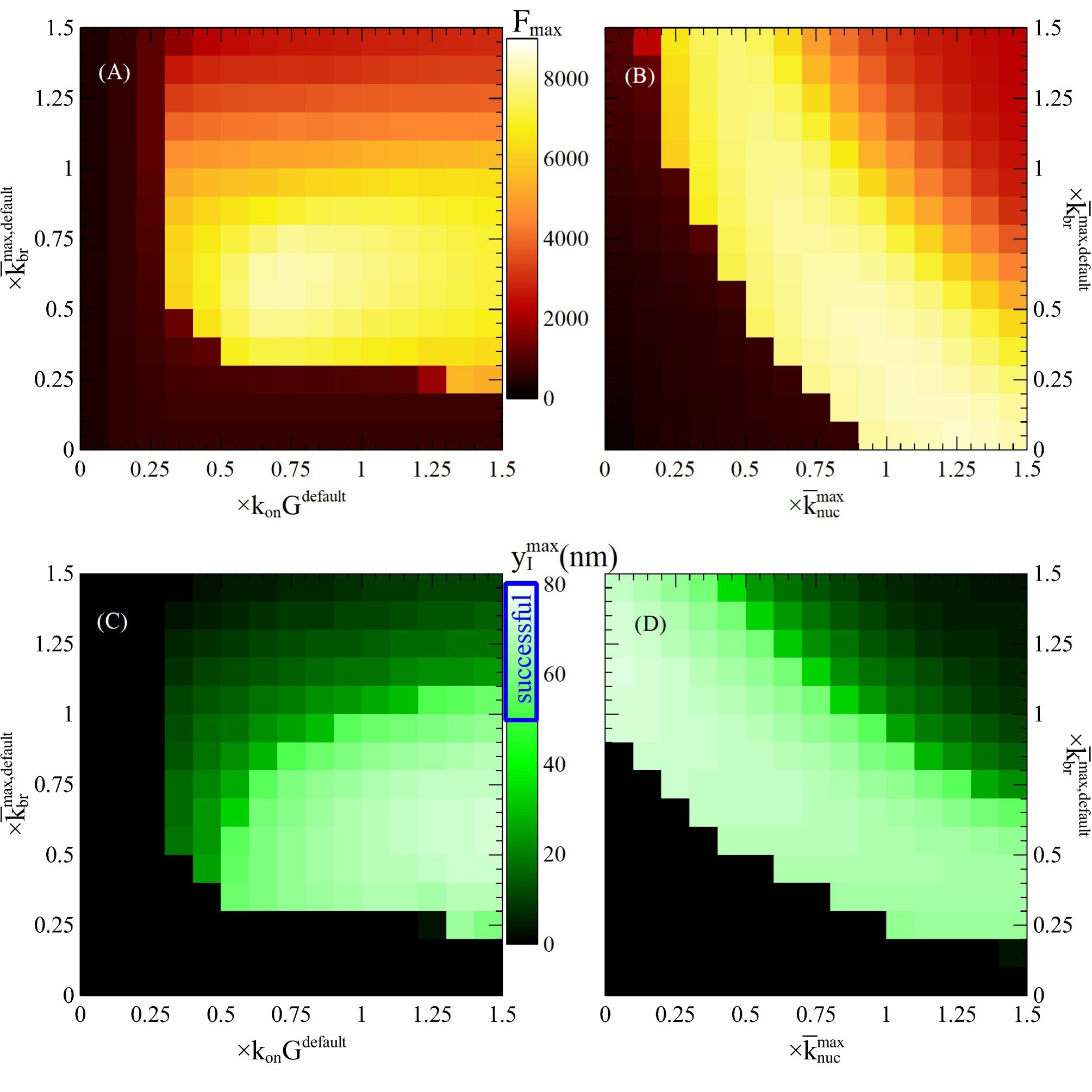

Supplement: S4 Fig — (A) Fmax vs. k¯brmax and kon Gdefault. (B) Fmax vs. k¯brmax and k¯nucmax. (C) yImax vs. k¯brmax and kon Gdefault. (D) yImax vs. k¯brmax and k¯nucmax. Effects of breaking of pulling filaments occurring at small k¯nucmax are not included. (TIF) [file pcbi.1005901.s005.tif]
