# Supplementary material for: A master equation approach to actin polymerization applied to endocytosis in yeast
Source: PLoS Comput Biol. 2017 Dec 14;13(12):e1005901. doi: 10.1371/journal.pcbi.1005901 (PMC5746272; doi:10.1371/journal.pcbi.1005901)
Supplement: S1 Table — (PDF) [file pcbi.1005901.s006.pdf]

Parameter/Function	Meaning / Source or Estimation Method	Value
k_0 (parameter)	Autocatalytic assembly rate of Las17 / fitting	See Table 1
$k_{br}(r, y)$ (function)	Branching rate of the F-actin filaments / main text	-
$l(y)$ (function)	Length of newly branched F-actin filaments / main text	-
k_{sev} (parameter)	Severing rate of the F-actin filaments / fitting	Table 1
α (parameter)	Detachment rate of Las17 caused by branching / fitting	Table 1
$k_{nuc}(r, y)$ (function)	Nucleation rate / main text	-
N_{full} (parameter)	Maximum number of Las17 molecules / [6]	100
F_{min} (parameter)	F-actin threshold / Supplementary Material 10	500
a (parameter)	F-actin polymerization step size / [7]	2.7nm
k_{hmc} (parameter)	Spring constant in the actin force f_{out} / Supplementary Material 10	$0.5pN/a$
$1/\gamma$ (parameter)	Friction constant / Supplementary Material 10	$1.0pN^{-1} \times a$
r_{nuc} (parameter)	Limit of spontaneous nucleation layer / Supplementary Material 10	$10a$
y_{nuc} (parameter)	Limit of spontaneous nucleation layer / Supplementary Material 10	$10a$
y_{br} (parameter)	Limit of branching layer / [6]	$20a$
r_L^{in} (parameter)	Inner radius of Las17 ring / [6]	$10a$
r_L^{out} (parameter)	Outer radius of Las17 ring / [6]	$30a$
σ_{nuc} (parameter)	Gaussian width in $k_{nuc}(r, y)$ / Supplementary Material 10	r_L^{in}
σ_{br} (parameter)	Gaussian height in $k_{br}(r, y)$ / Supplementary Material 10	$0.5y_{br}$
κ (parameter)	Bending modulus of membrane / [1]	$328k_BT$
Π (parameter)	Turgor pressure / [3]	$0.2MPa$
R_Π (parameter)	Length scale / [1]	$15nm$
C_0 (parameter)	Spontaneous curvature / [1]	$0.4/R_\Pi$
σ (parameter)	Membrane tension / [1]	0
